# Identification of High-Temperature Tolerant Lentil (*Lens culinaris* Medik.) Genotypes through Leaf and Pollen Traits

**DOI:** 10.3389/fpls.2017.00744

**Published:** 2017-05-19

**Authors:** Kumari Sita, Akanksha Sehgal, Jitendra Kumar, Shiv Kumar, Sarvjeet Singh, Kadambot H. M. Siddique, Harsh Nayyar

**Affiliations:** ^1^Department of Botany, Panjab UniversityChandigarh, India; ^2^Indian Institute of Pulses ResearchKanpur, India; ^3^International Center for Agricultural Research in the Dry AreasRabat, Morocco; ^4^Department of Plant Breeding and Genetics, Punjab Agricultural UniversityLudhiana, India; ^5^The UWA Institute of Agriculture, The University of Western AustraliaPerth, WA, Australia

**Keywords:** high temperature, reproductive growth, screening, tolerance, mechanisms, antioxidants

## Abstract

Rising temperatures are proving detrimental for various agricultural crops. Cool-season legumes such as lentil (*Lens culunaris* Medik.) are sensitive to even small increases in temperature during the reproductive stage, hence the need to explore the available germplasm for heat tolerance as well as its underlying mechanisms. In the present study, a set of 38 core lentil accessions were screened for heat stress tolerance by sowing 2 months later (first week of January; max/min temperature >32/20°C during the reproductive stage) than the recommended date of sowing (first week of November; max/min temperature <32/20°C during the reproductive stage). Screening revealed some promising heat-tolerant genotypes (IG2507, IG3263, IG3297, IG3312, IG3327, IG3546, IG3330, IG3745, IG4258, and FLIP2009) which can be used in a breeding program. Five heat-tolerant (HT) genotypes (IG2507, IG3263, IG3745, IG4258, and FLIP2009) and five heat-sensitive (HS) genotypes (IG2821, IG2849, IG4242, IG3973, IG3964) were selected from the screened germplasm and subjected to further analysis by growing them the following year under similar conditions to probe the mechanisms associated with heat tolerance. Comparative studies on reproductive function revealed significantly higher pollen germination, pollen viability, stigmatic function, ovular viability, pollen tube growth through the style, and pod set in HT genotypes under heat stress. Nodulation was remarkably higher (1.8–22-fold) in HT genotypes. Moreover, HT genotypes produced more sucrose in their leaves (65–73%) and anthers (35–78%) that HS genotypes, which was associated with superior reproductive function and nodulation. Exogenous supplementation of sucrose to *in vitro*-grown pollen grains, collected from heat-stressed plants, enhanced their germination ability. Assessment of the leaves of HT genotypes suggested significantly less damage to membranes (1.3–1.4-fold), photosynthetic function (1.14–1.17-fold) and cellular oxidizing ability (1.1–1.5-fold) than HS genotypes, which was linked to higher relative leaf water content (RLWC) and stomatal conductance (*gS*). Consequently, HT genotypes had less oxidative damage (measured as malondialdehyde and hydrogen peroxide concentration), coupled with a higher expression of antioxidants, especially those of the ascorbate–glutathione pathway. Controlled environment studies on contrasting genotypes further supported the impact of heat stress and differentiated the response of HT and HS genotypes to varying temperatures. Our studies indicated that temperatures >35/25°C were highly detrimental for growth and yield in lentil. While HT genotypes tolerated temperatures up to 40/30°C by producing fewer pods, the HS genotypes failed to do so even at 38/28°C. The findings attributed heat tolerance to superior pollen function and higher expression of leaf antioxidants.

## Introduction

Global temperatures would probably increase significantly by the end of the twenty-first century due to anthropogenic activities that will increase gases particularly carbon dioxide, methane, chlorofluorocarbons, and nitrous oxides (Sánchez et al., 2014). This will have devastating influence on the growth and development of plants, further reducing their potential yield and quality of food products (Delahunty et al., 2015). Moreover, local increases in temperature are higher than the global level, and more damaging to crops grown in these regions (Kaushal et al., 2013; Kaur et al., 2015). The elevated temperatures, particularly in tropical and subtropical regions, are markedly affecting the growth and yield of various winter and summer-season crops. These crops need to be examined as to how heat stress affects their vegetative and reproductive growth stages involving various morpho-physiological approaches. Various studies on on legumes such as chickpea (*Cicer arietinum* L.; Devasirvatham et al., 2012; Kaushal et al., 2013; Kumar et al., 2013), pea (*Pisum sativum L*.; Guilioni et al., 1997), common bean (*Phaseolus vulgaris L*.; Gross and Kigel, 1994), mung bean (*Vigna radiata* L.; Tzudir, 2014) and cowpea (*Vigna unguiculata L.;* Ahmed et al., 1992) have reported adverse effects of heat stress. Similar studies on lentil are limited (Delahunty et al., 2015; Bhandari et al., 2016; Kumar et al., 2016), where susceptibility of vegetative growth and reproductive function to heat stress has been depicted. Hence, further investigation is needed involving a large number of contrasting genotypes grown under similar heat stress environment to understand the mechanism of heat tolerance in this crop.

Lentil is sown as a cool-season crop, and is highly susceptible to rising temperatures. It needs low temperatures at the time of vegetative growth, while maturity requires warm temperatures; the best temperature for its optimum growth has been found to be 18–30°C (Sinsawat et al., 2004; Roy et al., 2012). Lentil is also grown in relatively warmer regions in central and southern parts of India, where the crop is exposed to supra-optimal temperatures that reduce its yield potential (Verma et al., 2014). Moreover, it has been observed that the chilling periods are becoming shorter and the heat periods are becoming longer, further resulting in exposure of cool-season crops to heat stress, particularly in the reproductive stage (Hasanuzzaman et al., 2013). Heat stress of 35°C was vital in discriminating the heat-tolerant and heat-sensitive genotypes in chickpea (*Cicer arietinum* L.) and faba bean (*Vicia faba* L.) (Gaur et al., 2015). Temperatures above 32/20°C (max/min) during flowering and pod filling in lentil can drastically reduce seed yield and quality (Delahunty et al., 2015). In 2009, across southeastern Australia, a heat wave (35°C for 6 days) reduced the yield in lentil crops by 70% (Delahunty et al., 2015).

Heat stress can affect the growth, development, metabolism and productivity of plants (Hasanuzzaman et al., 2013). Heat stress causes various physiological changes in plants such as leaf and stem scorching, leaf abscission and senescence, shoot and root growth inhibition, reduction in the number of flowers, inhibited pollen tube growth, pollen infertility, and fruit damage, leading to catastrophic losses in crop yields (Bita and Gerats, 2013; Teixeira et al., 2013; Hemantaranjan et al., 2014). Above-normal temperatures also affect membrane stability, water relations, photosynthesis, respiration and modulate the concentration of hormones, and primary and secondary metabolites (Hemantaranjan et al., 2014). In leaves, the process of photosynthesis is recognized as susceptible to high temperatures and may get retarded because of chlorosis, impaired electron flow, thermolability of photosystem II (PSII), and decreased carbon fixation as well as assimilation (Sinsawat et al., 2004).

Reproductive development (flowering and seed filling) is most susceptible to high temperature stress; and rise in temperature during flowering by a few degrees can lead to complete crop loss (Wheeler et al., 2000; Asseng et al., 2011; Hatfield, 2011). “At the time of reproduction, a brief phase of high temperature may decrease the number of floral buds and augment abortion of flowers abortion, significantly, though variations occur in the response within and amid plant species as well as their genotypes” (Annisa et al., 2013; Kaushal et al., 2013; Sage et al., 2015). Investigations involving exposure to moderate heat stress at various reproductive stages have associated development and performance of pollen grains as being highly susceptible to elevated temperature stress (Kaushal et al., 2013; Jiang et al., 2015; Sage et al., 2015). High temperatures may interrupt reproductive function by changing the concentrations of phytohormones like auxins (Teale et al., 2006) and abscisic acid (Todaka et al., 2012).

Heat stress also speeds up the production and reactions of reactive oxygen species (ROS) including singlet oxygen, superoxide, hydroxyl radical and hydrogen peroxide thereby inducing oxidative stress (Mittler, 2002; Hasanuzzaman et al., 2012), which can significantly damage cell structure (Chakraborty and Pradhan, 2012). Prolonged accumulation of ROS is harmful and can inactivate enzymes, lipid peroxidation, protein degradation and damage DNA (Chakraborty and Pradhan, 2012). The plants have several enzymatic and non-enzymatic systems, which detoxify the most toxic ROS to less reactive molecules to limit oxidative damage under heat stress (Sairam and Tyagi, 2004).

Enzymatic antioxidants, for example, peroxidase, catalase, superoxide dismutase, ascorbate peroxidase as well as glutathione reductase work for the removal of superoxides and hydrogen peroxide (Mittler, 2002). Non-enzymatic antioxidants like tocopherols, carotenoids, ascorbic acid, glutathione, also act along with enzymatic antioxidants against oxidative stress (Foyer and Noctor, 2003). Their higher expression has been linked with heat tolerance in some previous studies lentil (*Lens culinaris* Medik.; Chakraborty and Pradhan, 2011), soybean (*Glycine max* L.; Devi and Giridhar, 2015), pea (*Pisum sativum* L.; Osman, 2015).

The mechanisms affecting heat tolerance are not fully known in lentil. The objective of this study was to (a) screen the core lentil germplasm for heat tolerance and (b) understand the basis of heat tolerance using contrasting genotypes.

## Materials and methods

### Screening for heat tolerance

The seeds of 38 lentil (*Lens culinaris* Medik.) genotypes were procured from different sources (Punjab Agricultural University, Ludhiana, India; Indian Institute of Pulse Research, Kanpur, India; ICARDA, Morocco). The lentil genotypes were sown in earthen pots (8 kg capacity) on two sowing dates: (1) first week of November 2013 for normal sowing and (2) first week of January 2014 for late sowing to impose heat stress at the reproductive stage. For normal-sown plants, temperatures ranged from 27.3 to 6.1°C (maximum) and 15 to 3°C (minimum) during the vegetative stage and from 31.5 to 17.4°C (maximum) and 18.2 to 9.8°C (minimum) during the reproductive stage. For late-sown plants, temperatures ranged from 33 to 17.2°C (maximum) and 18.2 to 9.2°C (minimum) during the vegetative stage and from 38 to 27.5°C (maximum) and 28–14°C (minimum) during the reproductive stage.

Relative humidity for normal-sown plants ranged from 100 to 72% (maximum) and 82 to 21% (minimum) during the vegetative stage and from 95 to 77% (maximum) and 61 to 14% (minimum) during the reproductive stage. In late-sown plants, the relative humidity ranged from 97 to 61% (maximum) and 78 to 14% (minimum) during the vegetative stage and from 93 to 42% (maximum) and 59 to 12% (minimum) during the reproductive stage.

### Raising of contrasting genotypes

Based upon the response of the 38 lentil genotypes to heat stress in the 2013–2014 trial above, several genotypes varying in heat sensitivity (five heat-tolerant and five heat-sensitive) were selected for further studies. These genotypes were sown on two sowing dates: (1) 12 November 2014 for normal sowing and (2) 15 January 2015 for late sowing to ensure heat stress during reproductive growth. The plants were grown under natural outdoor environment at Panjab University, Chandigarh, India (30° 44′ 5.9994″ N, 76° 47′ 27.5994″ E). In northern India, lentils are normally sown in November, the temperatures throughout reproductive development remain below 32°C/20°C (day time maximum/night time minimum); sowing in January would ensure that plants were exposed to heat stress (above 32°C/20°C) at the time reproductive development. A sandy loam soil (sand: 63.4%, silt: 24.6%, clay:12%) was mixed with sand in a 3:1 ratio. The growth medium (soil) was prepared by adding one part farmyard manure to three parts of the soil–sand mixture. Ten mg kg^−1^ of tricalcium phosphate fertilizer was also added. The mixture was used to fill earthen pots (300 mm in diameter; 8 kg soil capacity) (Awasthi et al., 2014). For inoculating the seeds, lentil-specific *Rhizobium* spp. was applied prior to sowing. Initially, ten seeds were sown in each pot, upon emergence, plants were thinned to five per pot, 15 days after sowing (DAS).

#### Weather data

**Figure 1:**
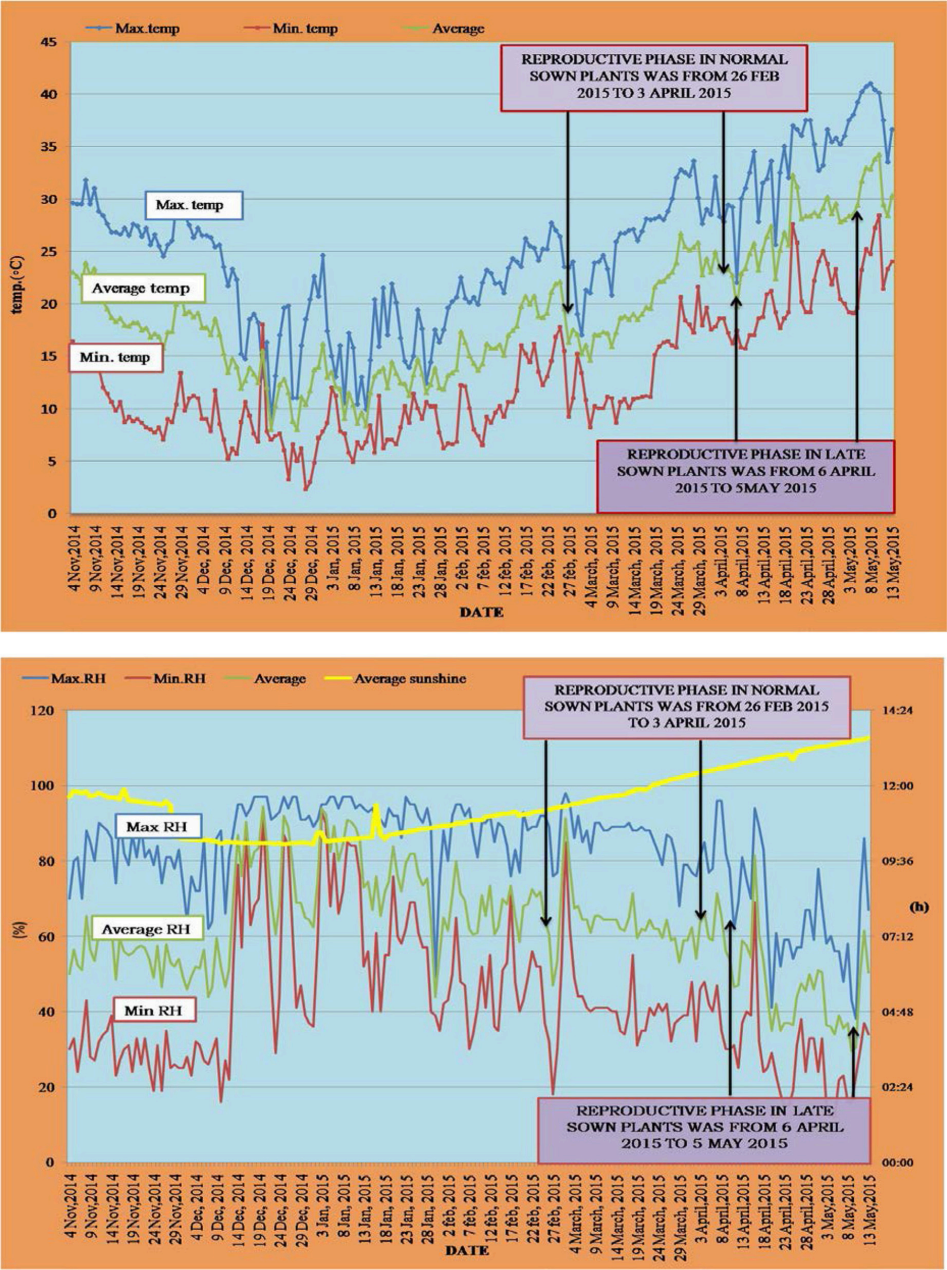
**Temperature profile (°C; top)** and RH (%) and photoperiod (hours; **bottom**) during the normal sown and late sown experiments. Arrows indicate the reproductive phase during both environments. Yellow line in the below figure represents the photoperiod during both sowing environments.

The day time maximum/night time minimum and mean air temperatures (Figure 1) and day time maximum/night time minimum and mean relative humidity (Figure 1) were recorded between 12 November 2014 and 5 May 2015. For normal-sown plants, temperatures ranged from 31.8 to 10°C (day time maximum) and 17.8 to 6.2°C (night time minimum) during the vegetative stage and from 33.6 to 17°C (day time maximum) and 21.6 to 8.2°C (night time minimum) during the reproductive stage. For late-sown plants, temperatures ranged from 33.6 to 17°C (day time maximum) and 20.6 to 9.2°C (night time minimum) during the vegetative stage and from 39.2 to 25.6°C (day time maximum) and 25 to 15.7°C (night time minimum) during the reproductive stage (Figure 1).

Relative humidity (RH) for normal-sown plants ranged from 97 to 49% (day time maximum) and 90 to 19% (night time minimum) during the vegetative stage and from 98 to 68% (day time maximum) and 62 to 18% (night time minimum) during the reproductive stage. In late-sown plants, the RH ranged from 98 to 68% (day time maximum) and 71 to 31% (night time minimum) during the vegetative stage, and from 96 to 41% (day time maximum) and 47 to 12% (night time minimum) during the reproductive stage (Figure 1).

Photoperiod ranged between 11.2 and 11.4 h under normal-sowing conditions, while under late-sown condtions, it ranged from 12.2 to 12.4 h (Figure 1).

Thermal accumulation units were 1,218.5 in normal-sown plants and 2,336.2 in late-sown plants. “Thermal units were calculated as the total of the average temperatures of all previous days until the initiation or completion of a particular stage” (Awasthi et al., 2014).

### Phenology, biomass, and yield components

The observations on phenology (days to flowering, podding and maturity), biomass, flower number, pod set (%), pod number and seed weight were recorded from 10 plants per genotype in three replications (30 plants/genotype). “Observations from replications for each genotype were pooled and averaged. The flowers were tagged and examined for pod set. Yield-related traits such as pod number, seed number and seed weight were also recorded at maturity. For yield data, mature seeds were collected, oven-dried for 3 days at 45°C and then weighed, with average values expressed on a per plant basis” (Awasthi et al., 2014).

### Reproductive biology

For the analysis of reproductive function from contrasting genotypes, flowers from individual plants (three per pot) were collected randomly from five pots (15 plants/genotype) in three replications from normal-sown and late-sown plants. The buds or flowers were harvested from normal-sown plants when the average temperature for the previous 7 days was lower than 25/19°C and from late-sown plants when the corresponding temperature was above 32/20°C. Floral biology was examined as follows:

#### Anther morphology

The morphology of anthers was evaluated using scanning electron microscopy (SEM). The fresh flowers were collected early in the morning on the day of anthesis, from control and stressed plants. Anthers of each genotype were collected from 10 flowers and put on a metallic stub. The anthers were observed under SEM to examine any structural changes. “Anthers were mounted fresh using double-stick tape, without dehydration, and critical point drying, sputter coated with gold paladium and scanned under SEM” (Postek et al., 1980; Kaushal et al., 2013).

#### Pollen morphology

“The morphology of pollen grains was studied using SEM. Pollen grains were removed from the anthers (collected as described above) and observed under SEM to examine any structural changes. On the day of anthesis, fresh flowers were collected early in the morning from control and stressed plants. Anthers from 10 flowers were collected and teased on a metallic stub. Samples were mounted fresh with double-stick tape, without dehydration, and critical point drying, sputter coated with gold paladium and scanned under SEM” (Postek et al., 1980; Kaushal et al., 2013).

#### Pollen viability

“About 200 pollen grains were tested for pollen viability was tested on 200 pollen grains (5–10 microscopic fields) with 0.5% acetocarmine/Alexander stain” (Kaushal et al., 2013). The pollen grains were collected from flowers, which opened on the same day. The pollen grains collected from flowers were pooled and tested for their viability (Alexander, 1969). To select viable pollen grains, section was made on the basis of size and shape (triangular or spherical) of the pollen, and the concentration of the stain taken up by the pollen (Kaushal et al., 2013).

#### Pollen load and pollen germination

“The pollen load and pollen germination (*in vivo*) was tested from the flowers (collected as above for pollen viability) with fully-dehiscent anthers and pollen grains on the stigma. Pollen load on the stigma was scored on a 1–5 scale (1 = low and 5 = high; Srinivasan et al., 1999). The number of germinated and ungerminated pollen grains on the stigma surface were counted from normal and heat-stressed flowers (Kaushal et al., 2013). The *in vitro* pollen germination was tested in pollen grains collected from five flowers per genotype in three replications. Pollen were germinated as per the method of Brewbaker and Kwack (1963) using a medium containing 10% sucrose, 1,640 mM boric acid, 1,269 mM calcium nitrate, 812 mM magnesium sulfate and 990 mM potassium nitrate (pH 6.5). Pollen grains were treated as germinated when the size of tube exceeded the diameter of the pollen grain. The percentage germination was determined from at least 100 pollen grains per replicate” (Kaushal et al., 2013).

#### Pollen germination (*in vivo*) and fate of pollen tube growth

“The pollen germination on the stigma and pollen tube growth through the style and in ovary was examined using Fluorescence microscopy. The flowers were collected 1–3 days after anthesis and fixed in acetic alcohol (1:3) for 24 h and then transferred to 8N NaOH for 6 h at 60°C for clearing purposes. The complete gynoecium part was transferred to aniline blue (0.1%), which was kept overnight, followed by mounting on a slide in a 1:1 (aniline blue: 10% glycerin) solution” (Kaushal et al., 2013). The stained gynoecium was observed under a fluorescence photomicrograph microscope (Nikon, Japan) (Dumas and Knox, 1983).

#### Stigma receptivity

“To detect stigma receptivity, an esterase test was carried out using a-naphthyl acetate as the substrate in the azo-coupling reaction with fast blue B, as modified by Mattson et al. (1974). Stigmas were removed from five flowers per genotype in three replications 1 day before flower opening, immersed in a solution containing a-naphthyl acetate and fast blue B in phosphate buffer, at 37°C for 15 min. The reddish brown color that developed on the surface of the stigma was scored on a 1–5 scale (1 = low receptivity and 5 = high receptivity)” (Kaushal et al., 2013).

#### Ovule viability

Ovule viability was assessed via a 2, 3, 5-triphenyl-2 H-tetrazolium chloride (TTC) reduction test. The ovules were carefully removed from the ovary of five flowers per genotype in three replications 1 day before anthesis. The ovules were placed into a drop of TTC solution (0.5% TTC in 1% sucrose solution) on a clean glass slide. The ovules were covered with a cover slip and kept in a petri-dish having moist filer paper (2 layers). The petri-dish was covered by a black paper and kept in the dark for incubation at 25°C in chamber. Following incubation of 15 min, the ovules were examined under the microscope for viability, which was measured on the basis of intensity of red color due to formazan formation, especially in the central region. The red color in the ovules is because of high availability of oxygen. The intensity of the red color (ovule viability) was scored on a 1–5 scale (1 = lowest intensity and 5 = highest intensity) (Kaushal et al., 2013).

To assess stress injury and biochemical traits, flowers and subtending leaves were harvested randomly from five pots (15 plants/genotype) in three replications from normal-sown and late-sown plants. The flowers or leaves were collected from normal-sown plants when the average temperature for the preceding 7 days was <25/19°C, and from late-sown plants (when the corresponding temperature was above 32/20°C). The following tests were conducted:

### Stomatal conductance and leaf temperature

Stomatal conductance and leaf temperature of top fully-expanded leaves were assessed with a portable leaf porometer (model SC1; Decagon Devices, Pullman, WA, USA) (Kaushal et al., 2013).

### Leaf photosynthetic function

“The photochemical efficiency of the intact leaves of the outdoor-grown pants was measured as chlorophyll fluorescence using the dark-adapted test of the modulated chlorophyll fluorometer OS1-FL (Opti-Sciences, Tyngsboro, MA, USA). With this system, chlorophyll fluorescence is excited by a 660 nm solid-state light source with filters blocking radiation longer than 690 nm. The average intensity of this modulated light was adjusted from 0 to 1 mE. Detection was in the 700–750 nm range using a PIN silicon photodiode with appropriate filtering to remove extraneous light. The clamps of the instrument were installed on the leaves to keep them in the dark and to stop the light reaction of photosynthesis for 45 min. After this, the clamps were attached to the optic fiber of the device and the valves of the clamps were opened. After starting the device, the 695 nm modulated light was radiated through the optic fiber toward the leaf. Subsequently, the Fv/Fm ratio was recorded. The leaves tested for chlorophyll fluorescence were also used for measurement of chlorophyll concentration” (Kaushal et al., 2013).

### Membrane damage (as electrolyte leakage)

The leaves subtending the flowers were harvested from plants grown under normal-sown and late-sown conditions (details as above). Electrolyte leakage was used to assess the permeability of the cell membrane as described by Lutts et al. (1996). Leaf segments after washing with deionized water were placed in closed vials containing deionized water (10 mL) and incubated overnight at 25°C. Electrical conductivity of the bathing solution (C_1_) was determined after 24 h. Samples were then put in a boiling water bath for 10–15 min and final conductivity reading (C_2_) was obtained upon equilibration at 25°C (Kaushal et al., 2013). The electrolyte leakage (EL) was defined as follows:

$$\begin{array}{l} \text{EL}\%={\text{C}}_{1}/{\text{C}}_{2}\times \;100 \end{array}$$

### Cellular oxidizing ability

The cellular oxidizing ability was measured as the 2, 3, 5-triphenyl tetrazolium chloride (TTC) reduction ability (Steponkus and Lanphear, 1967). One hundred mg of fresh leaf sample was cut into small strips, immersed in incubation solution (50 mM sodium phosphate, pH 7.4) containing TTC (500 mg 100 mL^−1^ solution) and incubated at 25°C in the dark. Since the TTC reduction is sensitive to excessive oxygen, the incubation of TTC was carried out without shaking. After two extractions with 95% ethanol (5 mL each), the extracts were pooled up to 10 mL. Formazan, which forms in green tissues, was measured at 530 nm instead of 485 nm to avoid intervention by pigments such as chlorophyll (Steponkus and Lanphear, 1967; Kaushal et al., 2013). The observations were expressed as absorbance per g of fresh weight (FW).

### Chlorophyll concentration

“To measure chlorophyll concentration, the fresh leaves (1.0 g) were homogenized in 80% acetone, followed by centrifugation at 5,701.8 g for 10 min. The absorbance of the supernatant was recorded at 645 and 663 nm, and total chlorophyll was calculated (Arnon, 1949) against 80% acetone as a blank” (Awasthi et al., 2014). The chlorophyll content was measured as:

$$\begin{array}{lll} \text{Chl a} & = & 12.9\;\left(\text{Ab}{\text{s}}_{663}\right)-2.69\;\left(\text{Ab}{\text{s}}_{645}\right)\;\frac{\text{V}}{1,000\times \text{ W}}\\ \text{Chl b} & = & 22.9\;\left({\text{Abs}}_{645}\right)-4.68\;\left({\text{Abs}}_{663}\right)\;\frac{\text{V}}{1,000\times \text{ W}}\\ \text{Total chl } & = & \text{ Chl a }+\text{Chl b} \end{array}$$

where V is volume, W is tissue weight, Abs_663_ is absorbance at 663 nm and Abs_645_ is absorbance at 645 nm. The total chlorophyll content was expressed as mg g^−1^ DW.

Fresh tissue was collected for measuring chlorophyll concentration and other biochemical traits but the calculations were made on dry weight (DW) basis. The dry weight of the fresh material was recorded by drying at 45°C for 2 days. The fresh weight was divided by the dry weight, the resultant vale was multiplied with the calculated units of any biochemical parameter.

### Relative leaf water content (RLWC)

RLWC was deterimined using the method of Barrs and Weatherley (1962). Leaf tissue (100 mg) was collected from control and stressed plants. The leaves were immersed in distilled water for 2 h in a petri dish, removed, followed by surface drying with blotters, and re-weighed (turgid weight, TW). The leaves were then oven-dried at 110°C for 24 h and weighed again for dry weight (DW) The relative leaf water content was calculated as follows:

$$\begin{array}{l} \text{Relative leaf water content }\left(\text{RLWC}\right)=\frac{\text{Fresh wt}-\text{Dry wt}.}{\text{Turgid wt}-\text{Dry wt}.}\times 10 \end{array}$$

### Soluble protein

The soluble protein concentration was estimated using a method devised by Lowry et al. (1951). The plant material (100 mg) was macerated in 0.1 M phosphate buffer (pH 7.0) and centrifuged at 513.16 g for 15 min to obtain a supernatant. Five mL of TCA (trichloroacetic acid; 15%) was added to the supernatant and kept at 4°C for 24 h. The mixture was then centrifuged at 513.16 g for 15 min to separate the precipitates. The supernatant was discarded and the precipitate dissolved in 0.1 N NaOH (1 mL), kept for 18 h for complete dissolution, and treated as an extract.

To 1 mL of the above extract, copper sulfate reagent containing 2% Na_2_CO_3_ (in 0.1 N NaOH) and 0.5% CuSO_4_.5H_2_O (in 1% sodium potassium tartrate) was added, allowed to stand for 15 min, before adding 0.5 mL of Folin-phenol Ciocalteu's reagent (1:1 ratio) (1 N). This mixture was kept for 30 min for color development before the absorbance was read at 570 nm. The total protein content (mg g^−1^ DW) was expressed using a standard curve plotted with bovine serum albumin.

### Sucrose

“The sucrose concentration was estimated as per the enzymatic method given by Jones et al. (1977). Leaf tissue was homogenized three times with 80% ethanol at 80°C for 1.5 h for each extraction. The extracts were pooled followed by evaporation at 40°C in an air-circulating oven. These were subsequently used to assay sucrose concentration. Aliquots of 200 μL from standard sucrose and the samples were added to 1 mL of reaction mixture consisting of imidazole buffer 100 mM (pH 6.9; 40 mM imidazole base, 60 mM imidazole-HCl), 0.4 mM NADP^+^, 1 mM ATP, 5 mM MgCl_2_, 0.5 mM dithiothreitol, 0.02% (w/v) bovine serum albumin (BSA), 20 μg mL^−1^ yeast invertase (E.C. 3.2.1.26), 2 μg mL^−1^ yeast hexokinase (E.C. 2.7.1.1) and 1 μg mL^−1^ yeast P-glucoisomerase (E.C. 5.3.1.9). The mixture was incubated at 25°C for 30 min to allow conversion of glucose and fructose to glucose 6-P. The absorption was meaured at 340 nm. Subsequently, 85 μL of glucose 6-P dehydrogenase (70 μl^−1^) was added, the mixture was re-read after ~5 min when absorbance became constant. Blanks were run with 200 μL of extract and 1 mL of the reaction mixture without invertase. The readings from each sample were changed to sucrose concentration with a standard curve” (Kaushal et al., 2013).

### Sucrose phosphate synthase

To assay this enzyme, enzymes, the leaf tissue was homogenized in a chilled HEPES buffer-NaOH (50 mM) pH 7 containing 2 mM MgCl_2_, 1 mM EDTA and 2 mM DTT as per the method of Déjardin et al. (1997). The desalting of the supernatant was done at 4°C by passing it through 4 mL Sephadex G-25 columns pre-equilibrated with buffer containing 20 mM HEPES–NaOH (pH 7.5), 0.25 mM MgCl_2_, 0.01% 2-mercaptoethanol, 1 mM ethylenediaminetetraacetic acid (EDTA) and 0.05% BSA. Sucrose phosphate synthase (SPS) activity was assayed from this extract following the anthrone test (Huber et al., 1989). During this process, 70 mL of the reaction mixture including the extract was adjusted to a final concentration of 4 mM Fru 6-P, 20 mM Glc 6-P, 3 mM UDPG, 50 mM HEPES–KOH (pH 7.5), 5 mM MgCl_2_ and 1 mM EDTA. The reaction mixture was incubated at 37°C for 15 min, and subsequently 70 mL of 30% (w/v) KOH was added followed by heating for 10 min at 95°C. To this, 1 mL of 0.14% (w/v) anthrone (in 95% H_2_SO_4_) was added. The mixture was incubated for 20 min at 37°C, and absorbance recorded at 650 nm. The sucrose concentration was calculated from the standard graph prepared with sucrose as the standard. The enzyme activity was expressed as μg sucrose min^−1^ mg^−1^ protein.

### Oxidative molecules

#### Malondialdehyde

The damage to membranes was assessed on the basis of a malondialdehyde (MDA) estimation, which indicates lipid peroxidation of the membranes, using the method described by Heath and Packer (1968). “Leaf tissue (1 g) was extracted in 10 mL of 0.1% trichloroacetic acid (TCA), followed by centrifugation at 11,319.75 g for 15 min. Subsequently, 4 mL of 0.5% thiobarbituric acid (in 20% trichloroacetic acid) was added to a 1-ml aliquot of the supernatant. The final volume was 5 ml. Thereafter, the mixture was heated for 30 min at 95°C, and rapidly cooled in an ice bath. After centrifugation at 5,701.8 g for 15 min, the absorbance was measured at 532 nm. The value for non-specific absorption at 600 nm was subtracted. MDA concentration was calculated using its absorption coefficient of 155 mmol^−1^ cm^−1^ and expressed as nmol g^−1^ DW” (Kaushal et al., 2013).

#### Hydrogen peroxide (H_2_O_2_)

The concentration of hydrogen peroxide (H_2_O_2_) was estimated using the method of Mukherji and Chaudhari (1983). Plant tissue (500 mg) was homogenized in 5 mL chilled acetone (80%) and filtered through Whatman filter paper 1. Four mL of titanium reagent was added followed by 5 mL of ammonia solution. The mixture was centrifuged at 5,031 g for 15 min and the supernatant was discarded. The residue was dissolved with 1 M H_2_SO_4_ and absorbance was recorded at 410 nm. The calculations were made with a standard curve plotted with pure H_2_O_2_ and expressed as μmol g^−1^ DW.

### Antioxidants (enzymatic and non-enzymatic)

#### Superoxide dismutase

The activity of superoxide dismutase (SOD; E.C. 1.15.1.1) was measured following the method of Dhindsa et al. (1981). Fresh plant tissue was homogenized in 50 mM chilled/ice cold phosphate buffer (pH 7.0) and centrifuged at 5,031 g for 15 min at 4°C and the supernatant was treated as an enzyme extract. The reaction mixture (3 mL) contained 13 mM methionine, 25 mM nitro-blue-tetrazolium (NBT), 0.1 mM EDTA, 50 mM sodium bicarbonate, 50 mM phosphate buffer (pH 7.8) and 0.1 mL of enzyme extract. The reaction was started by adding 2 mM riboflavin and exposing to 15 W fluorescent light for 10 min. The absorbance was recorded at 560 nm. SOD activity of the samples was assayed by recording its capacity to decrease the photochemical reduction of NBT. One unit of SOD activity was defined as the amount of enzyme which causes 50% inhibition of the photochemical reduction of NBT. It was expressed as Units mg^−1^ protein.

#### Catalase

Catalase (CAT; E.C. 1.11.1.6) activity was estimated using the method of Teranishi et al. (1974) with some modifications. The reaction mixture (3 mL) was prepared by mixing 50 mM phosphate buffer (pH 7.0), 200 mM H_2_O_2_ and 0.1 mL of enzyme extract. The reaction was initiated by adding 200 mM H_2_O_2_. The decrease in absorbance was recorded at 410 nm for 3 min. Catalase activity was measured using the extinction coefficient 40 mM^−1^ cm^−1^.

#### Ascorbate peroxidase

Ascorbate peroxidase (APO; E.C. 1.11.1.11) activity was determined by following the oxidation of ascorbate as a reduction in absorbance at 290 nm, using the method of Nakano and Asada (1981). Plant material was homogenized in ice cold 50 mM phosphate buffer, centrifuged at 5,031 g for 15 min at 4°C and the supernatant was kept for assay. The reaction was carried out at 20°C in 3 mL of reaction mixture containing 50 mM phosphate buffer (pH 7.0), 0.1 mM EDTA, 0.5 mM ascorbic acid and enzyme extract. Ascorbic acid (2 mM) was added to the reaction mixture to prevent inactivation of the enzyme. The change in A_290_ was recorded at 30 s intervals after addition of H_2_O_2_ for 3 min. The rate constant was calculated using the extinction coefficient of 2.8 mM^−1^ cm^−1^.

#### Glutathione reductase

Glutathione reductase (E.C. 1.6.4.2) was measured using the method of Mavis and Stellwagen (1968). The reaction mixture contained deionized water (0.65 mL), 100 mM phosphate buffer, pH 7.6 (1.5 mL), glutathione oxidized (GSSG; 0.1 mL), β-NADP (0.35 mL), BSA (0.20 mL) and enzyme solution (0.2 mL). The glutathione reductase enzyme solution was prepared immediately before use and contained 0.30–0.60 units mL^−1^ of glutathione reagent in cold reagent, i.e., 1% BSA. The contents of the reaction mixture were immediately mixed by inversion and the reduction in absorbance was read at 340 nm for approximately 3 min. The rate constant was calculated using the extinction coefficient of 6.2 mM^−1^ cm^−1^. The enzyme activity was expressed as mmol oxidized donor min^−1^ mg^−1^ protein.

#### Ascorbic acid

Ascorbic acid (AsA) was estimated according to the method of Mukherji and Chaudhari (1983). Plant tissue was homogenized in 6% TCA, and the homogenate was centrifuged at 3,649.15 g for 15 min. The supernatant was used as an extract for estimation. Two mL of 2% DNPH (Dinitrophenylhydrazine) was added to 4 mL of extract, followed by one drop of 10% thiourea. The mixture was boiled for 15 min in a water bath and then cooled to room temperature; subsequently 5 mL of chilled sulfuric acid was added at 0°C. The absorbance was read at 530 nm, and ascorbic acid concentration (mg g^−1^ DW) was calculated from a standard curve prepared by using known concentration ascorbic acid.

#### Glutathione

Reduced glutathione (GSH) was estimated following the method of Griffith (1980). “Fresh leaf tissue was homogenized in 2 mL of metaphosphoric acid followed by centrifugation at 14,539.59 g for 15 min. The aliquots of the supernatant were neutralized by adding 0.6 mL of 10% sodium citrate to 0.9 mL of the extract. A total volume of 1 mL of assay containing 700 μl NADPH (0.3 mM), 100 μl 5,5-dithio-bis-(2-nitrobenzoic acid (DTNB; 6 mM), 100 μl distilled water and 100 μl of the extract was prepared and allowed to stabilize at 25°C for 3–4 min. Thereafter, 10 μl of glutathione reductase (Sigma, USA) was added and the absorbance recorded at 412 nm” (Kaushal et al., 2013). Glutathione was calculated from a standard graph as described by Griffith (1980) and expressed as nmol g^−1^ DW.

#### Controlled environment studies

The contrasting genotypes of lentil (two heat-tolerant and two heat-sensitive) were grown outdoors and, at the onset of flowering (110–112 DAS) were subsequently subjected to controlled environment conditions at varying heat stresss [(35/25°C, 38/28°C, 40/30°C; day time/night time); (RH: 65–75% {day time}/45–55% {night time}] in a growth chamber (Meterx, New Delhi, India). Pod number and seed weight per plant were recorded. The plants were tested for reproductive function and damage to leaves using various tests.

#### Effect of sucrose on pollen germination (*in vitro*)

Pollen grains from heat-stressed flowers of two HT and two HS genotypes were collected and germinated in a controlled environment (method described above in reproductive biology) in a growth medium supplemented with 1.0 and 2.5 μM sucrose. The effects of sucrose concentration were tested on pollen germination.

#### Statistical analysis

There were 10 pots per genotype having 3 plants in 3 replications for screening experiments, which were randomized following RBD. For observations on contrasting genotypes too, 5 HT and 5 HS genotypes were grown using RBD under normal and late-won situations in 10 pots per genotype having 3 plants in 3 replications. For CE studies, 3 pots having 3 plants were used for each genotype using CRBD. Data were analyzed as two factorial (temperature and genotypes) experimental design using AGRISTAT statistical software (ICAR Research Complex, Goa, India). Standard errors and least significant differences (*P* < 0.05) for genotypes, treatments and their interaction were computed.

## Results

### Field experiment

In the initial experiment, 38 core accessions of lentil were screened for heat tolerance at the reproductive stage on the basis of biomass, filled pods per plant, seed weight per plant and 100-seed weight in 2013–2014.

Observations revealed that in normal-sown (NS) plants the biomass ranged from 3.1 to 7.5 g plant^−1^ across the screened genotypes, but only 1.0–3.1 g plant^−1^ in late-sown (LS) plants due to heat stress (Table 1). Among all the genotypes, IG3327, IG3312, IG3263, IG2507, IG3641, IG2458, IG4318, IG5146, and FLIP2009 produced the most biomass (2.6–3.1 g plant^−1^) under LS environment while IG2519, IG3568, IG4221, ILL6002, DPL15 produced the least (0.84–1.12 g plant^−1^).

**Table 1:** **Biomass and yield components in normal-sown (NS) and late-sown (LS) lentil genotypes (Mean ± SE)**.

	**Genotypes**	**Biomass g/plant**		**Filled pods/plant**		**Seed weight/plant**		**100 seed weight**	
		**NS**	**LS**	**NS**	**LS**	**NS**	**LS**	**NS**	**LS**
1	IG2506	4.3 ± 0.8	0.87 ± 0.18	38.9 ± 3.9	5.1 ± 1.3	1.51 ± 0.24	0.57 ± 0.16	1.22 ± 0.13	0.65 ± 0.11
2	IG2507	6.8 ± 0.9	2.9 ± 0.21	110 ± 6.6	69.7 ± 8.2	3.89 ± 0.78	2.31 ± 0.19	3.67 ± 0.32	2.3 ± 0.21
3	IG2510	5.4 ± 0.8	1.8 ± 0.13	49.4 ± 5.3	9.4 ± 1.9	1.81 ± 0.67	0.76 ± 0.16	1.61 ± 0.29	1.1 ± 0.17
4	IG2519	4.9 ± 0.7	0.81 ± 0.11	36.3 ± 4.8	7.3 ± 1.8	1.5 ± 0.34	0.61 ± 0.11	1.19 ± 0.28	0.49 ± 0.13
5	IG2802	5.8 ± 0.9	1.7 ± 0.13	41.5 ± 4.4	7.5 ± 1.9	1.83 ± 0.23	0.81 ± 0.13	1.65 ± 0.17	0.97 ± 0.15
6	IG2820	5.9 ± 0.9	1.8 ± 0.18	45.6 ± 4.8	9.1 ± 1.5	1.78 ± 0.22	0.88 ± 0.12	1.7 ± 0.20	1.13 ± 0.12
7	IG2821	6.1 ± 0.8	2.1 ± 0.15	83.5 ± 5.5	18.9 ± 2.2	3.88 ± 0.26	1.09 ± 0.14	1.97 ± 0.23	0.7 ± 0.11
8	IG2849	6.2 ± 0.9	2.3 ± 0.17	86.3 ± 6.1	20.4 ± 2.5	3.78 ± 0.27	0.92 ± 0.13	1.89 ± 0.24	0.54 ± 0.09
9	IG2878	5.7 ± 0.8	1.7 ± 0.13	41.4 ± 4.5	9.3 ± 1.3	1.76 ± 0.24	0.77 ± 0.14	1.66 ± 0.21	0.94 ± 0.17
10	IG3263	7.1 ± 0.9	3.1 ± 0.14	104 ± 5.8	62.3 ± 3.3	3.81 ± 0.25	2.46 ± 0.15	3.28 ± 0.24	2.18 ± 0.22
11	IG3290	5.8 ± 0.8	1.6 ± 0.16	44.3 ± 4.9	9.3 ± 1.4	1.71 ± 0.22	0.78 ± 0.15	1.63 ± 0.26	0.89 ± 0.21
12	IG3297	6.4 ± 0.8	2.3 ± 0.13	78.5 ± 5.9	30.5 ± 1.6	4.04 ± 0.87	2.12 ± 0.16	3.11 ± 0.24	1.56 ± 0.18
13	IG3312	7.3 ± 0.9	2.8 ± 0.24	111 ± 8.9	43.8 ± 1.5	4.32 ± 0.82	2.11 ± 0.19	3.46 ± 0.28	2.01 ± 0.23
14	IG3326	6.4 ± 0.8	2.5 ± 0.21	56.3 ± 6.3	24.6 ± 2.3	2.31 ± 0.36	1.78 ± 0.17	1.84 ± 0.26	0.94 ± 0.23
15	IG3327	7.2 ± 0.8	2.7 ± 0.19	121 ± 6.8	48.3 ± 2.3	4.14 ± 0.38	2.19 ± 0.18	3.44 ± 0.25	1.91 ± 0.29
16	IG3330	7.3 ± 0.9	2.1 ± 0.16	117 ± 8.8	49.2 ± 2.4	4.31 ± 0.42	2.16 ± 0.15	3.57 ± 0.36	1.87 ± 0.26
17	IG3364	5.3 ± 0.9	1.4 ± 0.14	53.2 ± 6.6	9.4 ± 1.8	1.79 ± 0.24	0.81 ± 0.14	1.68 ± 0.27	1.9 ± 0.29
18	IG3520	6.3 ± 0.9	2.7 ± 0.15	70.3 ± 8.2	26.7 ± 1.9	4.12 ± 0.33	2.01 ± 0.16	3.01 ± 0.22	1.49 ± 0.24
19	IG3537	6.1 ± 0.8	2.8 ± 0.16	73.4 ± 8.4	28.7 ± 1.5	4.08 ± 0.37	2.09 ± 0.17	3.09 ± 0.28	1.67 ± 0.28
20	IG3546	7.4 ± 0.8	2.5 ± 0.22	120 ± 9.8	43.9 ± 2.2	4.11 ± 0.38	2.11 ± 0.21	3.61 ± 0.43	2.01 ± 0.26
21	IG3568	4.8 ± 0.8	0.84 ± 0.13	35.6 ± 6.8	6.1 ± 1.3	1.59 ± 0.28	0.65 ± 0.14	1.17 ± 0.26	0.56 ± 0.19
22	IG3641	6.2 ± 0.8	2.9 ± 0.21	70.3 ± 7.3	27.3 ± 1.8	4.06 ± 0.78	2.14 ± 0.18	2.98 ± 0.48	1.73 ± 0.22
23	IG3745	7.5 ± 0.9	2.6 ± 0.23	127 ± 9.5	74.5 ± 5.4	3.79 ± 0.82	2.49 ± 0.16	3.79 ± 0.62	2.21 ± 0.24
24	IG3803	6.4 ± 0.9	2.5 ± 0.22	76.2 ± 8.5	26.4 ± 3.4	4.14 ± 0.88	2.19 ± 0.23	3.14 ± 0.48	1.67 ± 0.19
25	IG3964	5.14 ± 0.8	1.45 ± 0.11	78.4 ± 9.4	28.7 ± 3.5	2.98 ± 0.63	0.89 ± 0.18	2.34 ± 0.43	0.819 ± 0.22
26	IG3973	5.4 ± 0.8	1.13 ± 0.10	80.4 ± 8.3	30.7 ± 2.5	3.01 ± 0.71	0.95 ± 0.16	2.67 ± 0.35	1.04 ± 0.19
27	IG3984	6.6 ± 0.8	2.6 ± 0.24	66.4 ± 9.2	28.9 ± 3.5	4.11 ± 0.91	2.11 ± 0.24	3.06 ± 0.56	1.71 ± 0.21
28	IG4221	4.9 ± 0.8	0.78 ± 0.21	41.3 ± 6.4	9.4 ± 1.4	1.67 ± 0.23	0.72 ± 0.18	1.59 ± 0.34	0.93 ± 0.18
29	IG4242	5.4 ± 0.8	1.78 ± 0.18	81.7 ± 5.9	24.6 ± 2.5	3.67 ± 0.31	1.11 ± 0.17	1.79 ± 0.44	0.71 ± 0.19
30	IG4258	6.9 ± 0.7	2.4 ± 0.22	103 ± 8.9	60.6 ± 5.5	3.86 ± 0.42	2.33 ± 0.19	3.46 ± 0.51	2.04 ± 0.21
31	IG4318	6.2 ± 0.9	2.68 ± 0.25	80.3 ± 7.4	34.5 ± 4.3	4.02 ± 0.46	2.13 ± 0.21	3.13 ± 0.48	1.77 ± 0.22
32	IG5146	6.6 ± 0.8	2.72 ± 0.28	98 ± 8.3	48.6 ± 3.6	4.57 ± 0.48	2.21 ± 0.18	3.6 ± 0.51	2.11 ± 0.24
33	FLIP2009	6.8 ± 0.8	2.4 ± 0.22	103 ± 6.3	48.9 ± 3.2	3.83 ± 0.51	2.3 ± 0.18	3.85 ± 0.46	2.48 ± 0.25
34	DPL58	6.9 ± 0.9	2.4 ± 0.24	91.4 ± 8.1	40.3 ± 3.9	4.61 ± 0.66	2.16 ± 0.19	3.57 ± 0.54	2.05 ± 0.21
35	DPL315	5.2 ± 0.6	1.67 ± 0.19	40.3 ± 4.5	19.4 ± 3.5	2.17 ± 0.35	0.95 ± 0.16	1.81 ± 0.51	0.85 ± 0.18
37	DPL15	4.8 ± 0.6	1.1 ± 0.11	42.4 ± 3.3	17.4 ± 2.4	2.33 ± 0.45	0.91 ± 0.18	1.92 ± 0.43	0.84 ± 0.18
37	ILL-6002	3.04 ± 0.7	0.985 ± 0.14	25.3 ± 2.3	13.4 ± 1.5	1.13 ± 0.24	0.82 ± 0.17	0.96 ± 0.21	0.77 ± 0.19
38	DPL-15	4.7 ± 0.7	1.12 ± 0.12	48.6 ± 2.7	31.6 ± 2.6	2.23 ± 0.27	1.11 ± 0.20	2.01 ± 0.38	0.89 ± 0.17
LSD (*P* < 0.05; genotype × date of sowing Interaction)		0.84		3.8		0.32		0.25	

*LSD, least significant difference*.

Late-sown plants produced fewer filled pods (5.1–54.4 plant^−1^) due to heat stress than NS plants (25–127 plant^−1^) (Table 1). Under LS conditions, the genotypes IG2507, IG3263, IG3312, IG3327, IG3330, IG3546, IG3745, IG4258, IG5146, and FLIP2009 produced the most filled pods (43–54.4 plant^−1^) while IG2506, IG2510, IG2519, IG2802, IG2820, IG2878, IG3290, IG3364, and IG4221 produced the least (5.1–9.4 plant^−1^).

Seed weight in NS plants ranged from 1.13 to 4.61 g plant^−1^ while LS plants ranged from 0.57 to 2.49 g plant^−1^ (Table 1). In LS plants, genotypes IG2507, IG3263, IG3297, IG3312, IG3327, IG3330, IG3745, and FLIP2009 produced the heaviest seeds (2.13–2.49 g plant^−1^) while IG2506, IG2510, IG2802, IG2820, IG2878, IG3290, IG3364, IG3568, IG4221, ILL6002, DPL15, and DPL315 produced the lightest seeds (0.57–0.91 g plant^−1^).

In NS plants, 100-seed weight (Table 1) ranged from 0.96 to 3.85 g plant^−1^ but only 0.49–2.46 g plant^−1^ in LS plants. Genotypes IG2507, IG3263, IG3312, IG3546, IG4258, FLIP2009, and DPL58 had the highest 100-seed weights (2.04–2.46 g plant^−1^) while IG2506, IG2519, IG2802, IG2878, IG3290, IG3326, IG3568, IG4221, IG4242, DPL315, and DPL15 had the lowest (0.64–0.97 g plant^−1^).

Based upon this screening, genotypes IG2507, IG3263, IG3297, IG3312, IG3327, IG3546, IG3330, IG3745, IG4258, and FLIP2009 were relatively tolerant to heat stress, whereas genotypes IG2506, IG2519, IG2802, IG2821, IG2849, IG2878, IG3290, IG3326, IG3568, IG3973, IG3964, IG4221, IG4242, DPL315, and DPL15 were relatively heat-sensitive.

Of the 38 genotypes screened for heat tolerance, the five most-tolerant (IG2507, IG3263, IG3745, IG4258, and FLIP2009) and the five most-sensitive (IG2821, IG2849, IG4242, IG3973, IG3964) to heat stress were selected for further studies to probe the possible mechanisms related to heat tolerance. These genotypes were subsequently grown in 2014–2015 and tested for growth, yield, stress injury to leaves, and reproductive function using various tests. The weather parameters (temperature, RH and photoperiod) are detailed in the Materials and Methods (Figure 1).

### Growth and yield

Flowering occurred in 108–112 DAS in NS plants and 51.2–53.4 DAS in LS plants (Table 2). Podding took 112–116 days to initiate in NS plants and 54–59 days in LS plants (Table 2). The NS plants matured in 140–145 days while the LS plants matured in 91–94 days (Table 2).

**Table 2:** **Phenology of various genotypes under normal-sown (NS) and late-sown (LS) plants (Mean ± SE); T, heat-tolerant, S, heat-sensitive**.

**Genotypes**	**Days to flowering**		**Days to podding**		**Days to maturity**	
	**NS**	**LS**	**NS**	**LS**	**NS**	**LS**
IG2507 (T)	106 ± 3.1a	53.2 ± 2.4a	111 ± 2.6a	55 ± 1.5a	141 ± 4.6a	91 ± 6.9a
IG3263(T)	109 ± 2.8a	51.3 ± 2.3a	112 ± 2.8a	57 ± 1.9a	143 ± 5.1a	90 ± 8.9a
IG3745(T)	108 ± 2.5a	52.4 ± 2.1a	114 ± 2.4a	58 ± 1.5a	140 ± 4.5a	94 ± 9.2a
IG4258(T)	108 ± 2.4a	53.2 ± 2.3a	116 ± 2.9a	54 ± 1.6a	142 ± 5.6a	92 ± 8.4a
FLIP2009(T)	109 ± 2.9a	51.2 ± 2.4a	112 ± 2.4a	56 ± 1.8a	142 ± 5.8a	91 ± 9.1a
IG2821(S)	107 ± 2.3a	53.3 ± 2.2a	113 ± 2.1a	57 ± 1.4a	141 ± 5.9a	93 ± 6.5a
IG2849(S)	109 ± 2.5a	52.3 ± 2.5a	111 ± 2.0a	59 ± 1.3a	140 ± 4.9a	94 ± 5.8a
IG4242(S)	108 ± 2.3a	53.4 ± 2.3a	112 ± 2.3a	56 ± 1.5a	142 ± 8.5a	92 ± 5.9a
IG3973 (S)	107 ± 2.2a	51.3 ± 2.8a	114 ± 2.5a	58 ± 1.4a	140 ± 9.3a	91 ± 4.9a
IG3964 (S)	109 ± 2.8a	52.2 ± 2.1a	113 ± 2.2a	56 ± 1.6a	143 ± 8.8a	93 ± 5.1a
LSD (*P* < 0.05; genotype × date of sowing Interaction)	3.4		2.9		3.3	

*Similar letters in a vertical column indicate no significant difference from each other. LSD, least significant difference*.

The flowering duration in LS plants was shorter than in NS plants; by 10–11 days in heat-tolerant (HT) genotypes and 14–18 days in heat-sensitive (HS) genotypes (Table 3). Similarly, the podding duration was 9–11 days shorter in HT genotypes and 16–19 days in HS genotypes (Table 3).

**Table 3:** **Flowering and podding duration, number of flowers and pod set (%) in normal-sown (NS) and late-sown (LS) plants of heat-tolerant (T) and heat-sensitive (S) genotypes (Mean ± SE)**.

**Genotypes**	**Flowering duration (days)**		**Podding duration (days)**		**Number of flowers**		**Pod set (%)**	
	**NS**	**LS**	**NS**	**LS**	**NS**	**LS**	**NS**	**LS**
IG2507 (T)	28 ± 2.3a	18 ± 2.7a	30 ± 2.6a	20 ± 1.5a	123 ± 4.6ab	78 ± 6.9a	89.4 ± 6.8a	78.4 ± 8.4a
IG3263(T)	30 ± 3.1a	20 ± 2.1a	28 ± 2.8a	19 ± 1.9a	118 ± 5.1ab	71 ± 8.9a	88.4 ± 8.7a	77.7 ± 8.3a
IG3745(T)	31 ± 2.3a	20 ± 1.9a	30 ± 2.4a	20 ± 1.5a	140 ± 4.5a	82 ± 9.2a	90.7 ± 9.3a	71.2 ± 8.1a
IG4258(T)	29 ± 2.5a	18 ± 2.5a	30 ± 2.9a	19 ± 1.6a	106 ± 5.6*c*	68 ± 8.4ab	84 ± 7.7a	76.1 ± 8.3a
FLIP2009(T)	30 ± 3.3a	20 ± 2.2a	29 ± 2.4a	18 ± 1.8a	131 ± 5.8a	69 ± 9.1ab	88.9 ± 8.4a	77.8 ± 8.2a
IG2821(S)	29 ± 2.5a	14 ± 1.1b	28 ± 2.1a	12 ± 1.4b	117 ± 5.9ab	37 ± 6.5*c*	71.3 ± 9.4b	41 ± 5.6b
IG2849(S)	31 ± 2.8a	13 ± 1.6b	29 ± 2.0a	11 ± 1.3b	124 ± 4.9ab	35 ± 5.8*c*	69.5 ± 8.6b	40 ± 4.5b
IG4242(S)	28 ± 2.6a	12 ± 1.7b	29 ± 2.3a	10 ± 1.5b	115 ± 8.5ab	40 ± 5.9*c*	71 ± 9.5b	38 ± 4.1b
IG3973 (S)	29 ± 2.3a	11 ± 1.3b	30 ± 2.5a	12 ± 1.4b	113 ± 9.3ab	48 ± 4.9*c*	71 ± 9.2b	32 ± 4.8b
IG3964 (S)	30 ± 3.2a	12 ± 1.2b	26 ± 2.2a	10 ± 1.6b	109 ± 8.8*c*	49 ± 5.1*c*	71.5 ± 8.9b	37 ± 4.3b
LSD (*P* < 0.05; genotype × date of sowing Interaction)	3.4		2.9		10.3		10.5	

*Similar letters in a vertical column indicate no significant difference from each other. LSD, least significant difference*.

Late-sown plants of HT genotypes produced 68–78 flowers per plant compared to 106–140 in NS plants (Table 3). On the other hand, late-sown plants of HS genotypes produced 35–49 flowers per plant compared to 109–117 in NS plants. Pod set in LS plants was 71–78% in HT genotypes and 32–41% in sensitive genotypes (Table 3).

Late-sown HT genotypes produced 47–53% less biomass than NS plants while HS genotypes showed produced 72–74% less (Table 4). Similarly, the number of filled pods was 35–40% less in late-sown HT genotypes and 60–66.8% less in HS genotypes than NS plants. Seed weight per plant was 36–41% less in HT genotypes and 55–70% less in HS genotypes. One-hundred seed weight was 33–41% less in HT genotypes and 61–70% less in HS genotypes. Genotype IG3745 produced the highest biomass, pods/plant and seed weight/plant while 100-seed weight was highest in FLIP2009 under heat stress environment (Table 4).

**Table 4:** **Yield components in normal-sown (NS) and late-sown (LS) plants of heat-tolerant (T) and heat-sensitive (S) genotypes (Mean ± SE)**.

**Genotypes**	**Biomass/plant**		**Filled pods/plant weight**		**Seed weight/plant**		**100 seed weight**	
	**NS**	**LS**	**NS**	**LS**	**NS**	**LS**	**NS**	**LS**
IG2507 (T)	6.4 ± 0.43a	3.07 ± 0.35a	110 ± 4.8b	69.7 ± 8.5a	3.89 ± 0.68a	2.31 ± 0.36a	3.67 ± 0.32a	2.3 ± 0.14a
IG3263(T)	6.9 ± 0.36a	3.2 ± 0.41a	104 ± 5.9b	62.3 ± 9.2b	3.81 ± 0.71a	2.46 ± 0.33a	3.28 ± 0.36c	2.18 ± 0.18b
IG3745(T)	7.2 ± 0.41a	3.8 ± 0.37a	127 ± 8.3a	74.5 ± 7.8a	3.79 ± 0.73a	2.49 ± 0.36a	3.79 ± 0.42a	2.21 ± 0.15b
IG4258(T)	7.3 ± 0.45a	3.2 ± 0.38a	90 ± 9.1c	58.6 ± 8.3b	3.86 ± 0.74a	2.33 ± 0.37a	3.46 ± 0.35b	2.04 ± 0.15b
FLIP2009(T)	7.1 ± 0.44a	3.4 ± 0.42a	103 ± 9.4b	60.6 ± 7.7b	3.83 ± 0.81a	2.3 ± 0.31a	3.85 ± 0.37a	2.46 ± 0.13a
IG2821(S)	6.7 ± 0.48a	1.9 ± 0.23b	83.5 ± 8.7c	18.9 ± 3.2c	3.21 ± 0.78a	1.09 ± 0.35b	1.97 ± 0.28f	0.7 ± 0.14cd
IG2849(S)	6.5 ± 0.54a	1.8 ± 0.35b	86.3 ± 9.1c	20.4 ± 2.7c	3.11 ± 0.81a	0.92 ± 0.23b	1.89 ± 0.21f	0.54 ± 0.10cd
IG4242(S)	6.1 ± 0.47a	1.6 ± 0.25b	81.7 ± 8.9c	24.6 ± 4.3c	3.26 ± 0.68a	1.11 ± 0.21b	1.79 ± 0.24f	0.71 ± 0.11cd
IG3973 (S)	5.9 ± 0.51ab	1.3 ± 0.28b	80.4 ± 9.3c	30.7 ± 3.1c	3.01 ± 0.63a	0.95 ± 0.27b	2.67 ± 0.23d	1.04 ± 0.12c
IG3964 (S)	6.2 ± 0.42a	1.8 ± 0.29b	78.4 ± 8.4cd	28.7 ± 2.8c	3.19 ± 0.71a	0.89 ± 0.18b	2.34 ± 0.25*e*	0.82 ± 0.12d
LSD (*P* < 0.05; genotype × date of sowing Interaction)	1.2		10.5		0.46		0.21	

*Similar letters in a vertical column indicate no significant difference from each other. LSD, least significant difference*.

The number of nodules ranged from 10.8 to 13.1 in NS plants (Table 5). Late-sown plants produced 38–44% fewer nodules in HT genotypes and 68–75% fewer in HS genotypes. HT genotypes IG4258 and IG3745 produced the most nodules (7.4) whereas HS genotypes IG3973 and IG2849 produced the least (2.9).

**Table 5:** **Number of nodules in in normal-sown (NS) and late-sown (LS) plants of heat-tolerant (HT) and heat-sensitive (HS) genotypes (Mean ± SE)**.

**Genotypes**	**Nodule number**	
	**NS**	**LS**
IG2507 (T)	11.3 ± 2.1a	6.4 ± 0.67a
IG3263(T)	12.5 ± 1.8a	6.9 ± 0.71a
IG3745(T)	13.1 ± 1.6a	7.2 ± 0.64a
IG4258(T)	12.1 ± 1.9a	7.4 ± 0.58a
FLIP2009(T)	11.8 ± 1.5a	6.8 ± 0.66a
IG2821(S)	10.8 ± 1.8a	3.4 ± 0.62b
IG2849(S)	12.4 ± 1.7a	2.9 ± 0.69b
IG4242(S)	12.6 ± 1.9a	3.6 ± 0.61b
IG3973 (S)	11.9 ± 1.7a	2.9 ± 0.71b
IG3964 (S)	12.3 ± 1.8a	3.1 ± 0.62b
LSD (*P* < 0.05; genotype × date of sowing Interaction)	2.1	

*Similar letters in a vertical column indicate no significant difference from each other. LSD, least significant difference*.

### Status of damage to leaves due to heat stress

Leaf temperatures ranged from 29 to 31°C in NS plants, while in LS plants, leaf temperatures varied from 32.9 to 34.7°C in HT genotypes and 37.5–38.7°C in HS genotypes Genotype FLIP2009 showed the lowest leaf temperature (32.9°C) under heat stress (Table 6).

**Table 6:** **Leaf temperature, stomatal conductance (***gS***) and relative leaf water content (RLWC) in normal-sown (NS) and late-sown (LS) plants of heat-tolerant (HT) and heat-sensitive (HS) genotypes (Mean ± SE)**.

**Genotypes**	**Leaf temperature (°C)**		**Stomatal conductance (gS) mmol/m^−2^/s^−1^**		**RLWC (%)**	
	**NS**	**LS**	**NS**	**LS**	**NS**	**LS**
IG2507 (T)	30.6 ± 0.35a	33.4 ± 0.23b	319.4 ± 9.7b	390.5 ± 10.9d	79.4 ± 3.2b	73.4 ± 3.3c
IG3263(T)	31.4 ± 0.23a	34.7 ± 0.18b	323.5 ± 11.3b	410.4 ± 12.5c	83.5 ± 4.2a	80.4 ± 4.1a
IG3745(T)	29.8 ± 0.21a	33.5 ± 0.17b	420.6 ± 12.6a	489.3 ± 12.5a	86.4 ± 2.9a	82.6 ± 3.8a
IG4258(T)	30.4 ± 0.24a	34.7 ± 0.20b	327.2 ± 13.5b	443.5 ± 11.9b	79.5 ± 3.1b	74.8 ± 3.5c
FLIP2009(T)	29.6 ± 0.22a	32.9 ± 0.19bc	421.1 ± 11.9a	497.5 ± 13.9a	82.3 ± 2.8a	78.4 ± 3.7b
IG2821(S)	30.9 ± 0.15a	38.4 ± 0.23a	317.4 ± 12.8b	221.3 ± 12.7f	82.5 ± 4.2a	63.2 ± 2.8d
IG2849(S)	31.5 ± 0.18a	37.4 ± 0.25a	319.4 ± 13.6b	205.6 ± 11.9f	80.4 ± 3.8b	65.1 ± 3.1d
IG4242(S)	29.6 ± 0.19a	37.6 ± 0.26a	424.5 ± 14.3a	313.5 ± 14.5e	83.4 ± 4.5a	62.8 ± 4.2d
IG3973 (S)	30.8 ± 0.20a	38.7 ± 0.21a	326.5 ± 12.3b	210.5 ± 13.8g	78.9 ± 3.7b	63.2 ± 3.6d
IG3964 (S)	29.7 ± 0.22a	37.5 ± 0.25a	320.5 ± 13.1b	224.5 ± 16.7f	81.3 ± 4.2b	64.2 ± 4.1d
LSD (*P* < 0.05; genotype × date of sowing Interaction)	1.3			19.5	3.9	

*Similar letters in a vertical column indicate no significant difference from each other. LSD, least significant difference*.

The stomatal conductance (*gS*) of leaves ranged from 319 to 424 mmol m^−2^ s^−1^ in NS plants. It was 205–313 mmol m^−2^ s^−1^ in LS plants of HS genotypes and 390–497 mmol m^−2^ s^−1^ in HT genotypes (Table 6). Genotype FLIP2009 had the highest *gS* in the LS environment (Table 6).

Leaf water status was measured using relative leaf water content (RLWC), which varied from 78.3 to 86.4% in NS plants (Table 6). Late-sown plants had lower RLWCs, but it was relatively higher in HT genotypes (73.4–82.6%) than HS genotypes (62–65%). A HT genotype IG4258 had the highest RLWC (74.6%) when late-sown, while an HS genotype (IG4242) had the lowest (62.8%).

Membrane damage was recorded as electrolyte leakage (EL), which ranged from 12.5 to 15.3% (Table 7). In LS plants, EL increased due to heat stress; HT genotypes had less damage (18.4–20.3%) than HS genotypes (21.3–25.5%). Genotypes IG3263 and FLIP2009 had the least damage while IG2821 and IG4242 had the most.

**Table 7:** **Electrolyte leakage, Photosystem (PS) II function, Chlorophyll (Chl) concentration and 2,3,5-Triphenyl tetrazolium chloride (TTC) reduction ability in normal-sown (NS) and late-sown (LS) plants of heat-tolerant (HT) and heat-sensitive (HS) genotypes (Mean ± SE)**.

**Genotypes**	**Electrolyte leakage (%)**		**PS II (Fv/Fm ratio)**		**Chlorophyll (mg/g DW)**		**TTC reduction ability (Absorbace_530_/g FW)**	
	**NS**	**LS**	**NS**	**LS**	**NS**	**LS**	**NS**	**LS**
IG2507 (T)	13.5 ± 2.5a	19.4 ± 2.6b	0.8 ± 0.04a	0.68 ± 0.06a	15.6 ± 1.9a	12.4 ± 1.9a	0.23 ± 0.03a	0.19 ± 0.03a
IG3263(T)	12.8 ± 2.9a	18.4 ± 2.1b	0.79 ± 0.03a	0.71 ± 0.07a	16.8 ± 2.1a	13.1 ± 1.7a	0.21 ± 0.04a	0.17 ± 0.03a
IG3745(T)	14.3 ± 2.6a	19.5 ± 1.8b	0.8 ± 0.05a	0.69 ± 0.05a	14.7 ± 1.8a	11 ± 1.7a	0.25 ± 0.05a	0.18 ± 0.02a
IG4258(T)	15.3 ± 2.8a	20.3 ± 1.6b	0.78 ± 0.04a	0.7 ± 0.06a	14.9 ± 1.7a	10.8 ± 1.6a	0.24 ± 0.03a	0.2 ± 0.02a
FLIP2009(T)	12.4 ± 1.8a	18.4 ± 1.5b	0.8 ± 0.05a	0.68 ± 0.08a	15.1 ± 1.8a	11.7 ± 1.8a	0.21 ± 0.03a	0.18 ± 0.02a
IG2821(S)	13.2 ± 2.5a	24.3 ± 2.2a	0.8 ± 0.06a	0.59 ± 0.08b	14.9 ± 1.9a	7.9 ± 1.3b	0.23 ± 0.04a	0.11 ± 0.03b
IG2849(S)	12.7 ± 2.4a	23.4 ± 2.8a	0.8 ± 0.05a	0.56 ± 0.07b	15.6 ± 1.7a	8.3 ± 1.5b	0.22 ± 0.05a	0.13 ± 0.02b
IG4242(S)	14.1 ± 2.8a	25.5 ± 2.9a	0.78 ± 0.05a	0.58 ± 0.08b	14.5 ± 1.7a	4.8 ± 1.2d	0.24 ± 0.03a	0.13 ± 0.02b
IG3973 (S)	12.5 ± 2.9a	21.3 ± 2.7a	0.79 ± 0.07a	0.52 ± 0.06b	16.3 ± 1.8a	9.3 ± 1.2b	0.21 ± 0.05a	0.12 ± 0.03b
IG3964 (S)	13.2 ± 2.5a	23.4 ± 2.6a	0.8 ± 0.08a	0.58 ± 0.08b	13.8 ± 1.6ab	6.9 ± 1.3*c*	0.24 ± 0.03a	0.11 ± 0.02b
LSD (*P* < 0.05; genotype × date of sowing Interaction)	3.1		0.084		1.92		0.04	

*Similar letters in a vertical column indicate no significant difference from each other. LSD, least significant difference; Fv, variable fluorescence; Fm, maximum fluorescence*.

Photosynthetic efficiency was measured as PSII function (Fv/Fm ratio). In LS plants, HS genotypes showed more reduction (21–26%) in PSII function than HT genotypes (10–15% reduction) (Table 7). A HT genotype IG3263 had the highest PSII function (0.71) while the HS genotype IG3964 had the lowest (0.58).

Stay-green trait was measured as the loss of total chlorophyll (chl) in leaves (Table 7). Total chlorophyll concentration in LS plants was lower (4.8–13.1 mg g^−1^ DW) than NS plants (13.8–15.6 mg g^−1^ DW). In LS plants, HT genotypes retained more total chlorophyll (10.8–13.1 mg g^−1^ DW) than HS genotypes (4.8–9.3 mg g^−1^ DW). Under heat stress, HT genotype IG3263 had the most chl (13.1 mg g^−1^ DW) while HS genotype IG4242 had the least (4.8 mg g^−1^ DW) chlorophyll.

Cellular oxidizing ability was measured in a TTC reduction assay (Table 7). Late-sown plants had significantly less cellular oxidizing ability (14–54%) than NS plants. HT genotypes maintained higher oxidizing ability values (0.17–0.2 units) than HS genotypes (0.11–0.13 units) under heat stress. Genotype IG2507 maintained highest respiration ability under hear stress.

**Figure 2:**
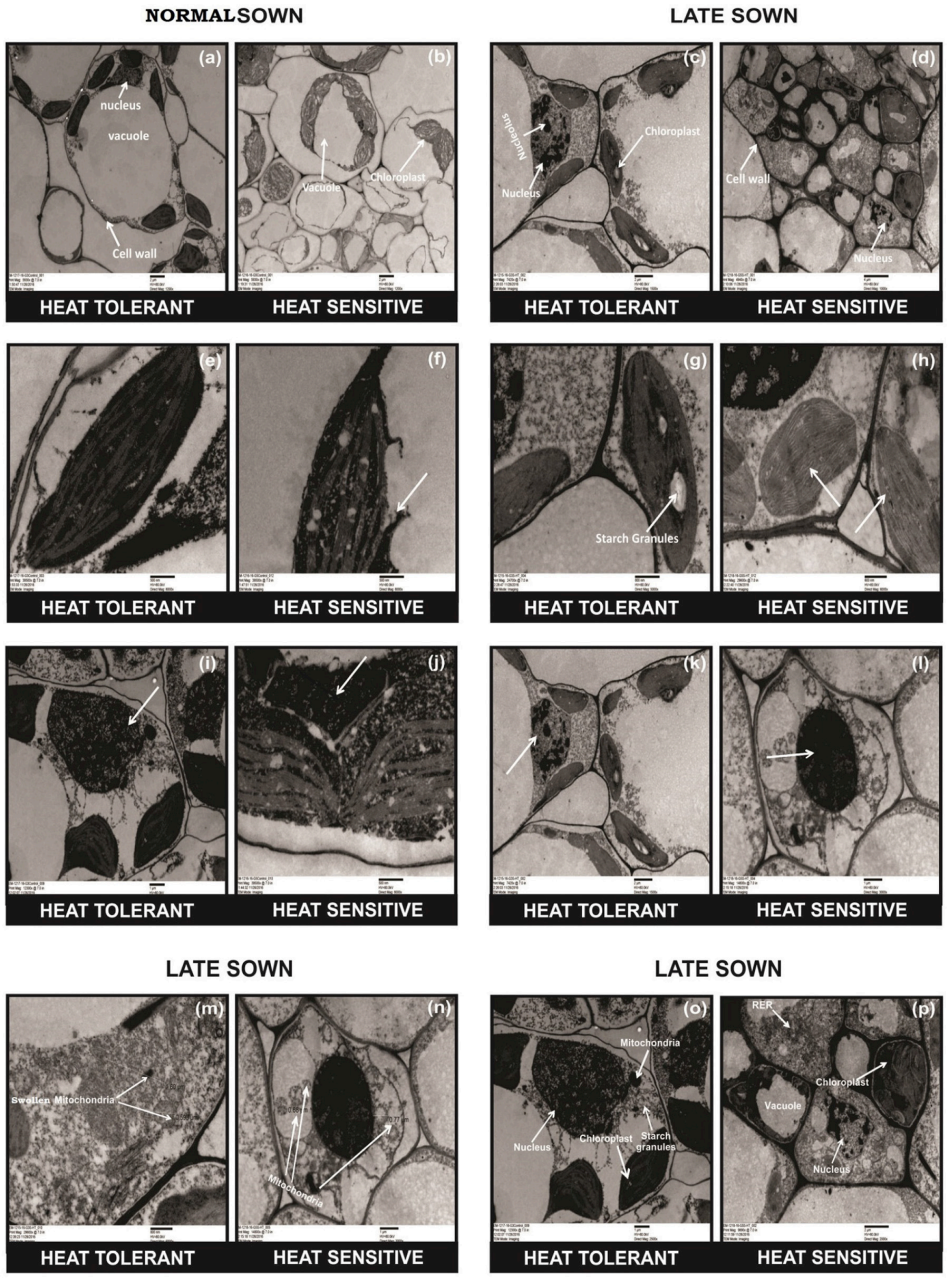
**Comparative TEM observations of the ultra-structure of leaves in normal sown (NS) and late sown (LS) plants**. Cell organelles from **(a,b)** NS and **(c,d)** LS plants; chloroplast from **(e,f)** NS and **(g,h)** LS plants; nucleus of **(i,j)** NS and **(k,l)** LS plants; mitochondria **(m,n)** from LS plants; cell wall **(o,p)** from LS plants. Arrows indicate **(b,d)** disrupted cell organelles, **(p)** thickened cell wall, **(f,h)** disrupted chloroplast, **(m,n)** damaged and increased number of mitochondria, **(p)** dispersed chromatin in nucleus, **(d,p)** shrinkage of vacuoles, and **(d,h)** fewer and smaller starch granules in the chloroplast in a heat-sensitive genotype under LS conditions.

TEM studies of leaves from heat-stressed plants showed cell wall thickening, severe damage to chloroplasts (as indicated by their shrinkage), fewer and smaller starch grains in the chloroplast, the disintegration of the chloroplast envelope, and damage to granal thylakoids, more so in HS genotypes (Figure 2, arrows). Late-sown HT genotypes had fewer mitochondria than late-sown HS genotypes Figure 2n arrows. The mitochondria also swelled under heat stress from (recorded as increase in size from 0.5 to 1.60 μm) as shown in Figures 2m,n arrows, while the nucleus contracted and the nucleus showed dispersed chromatin, with more damage to HS genotypes (Figure 2, arrows).

**Figure 3:**
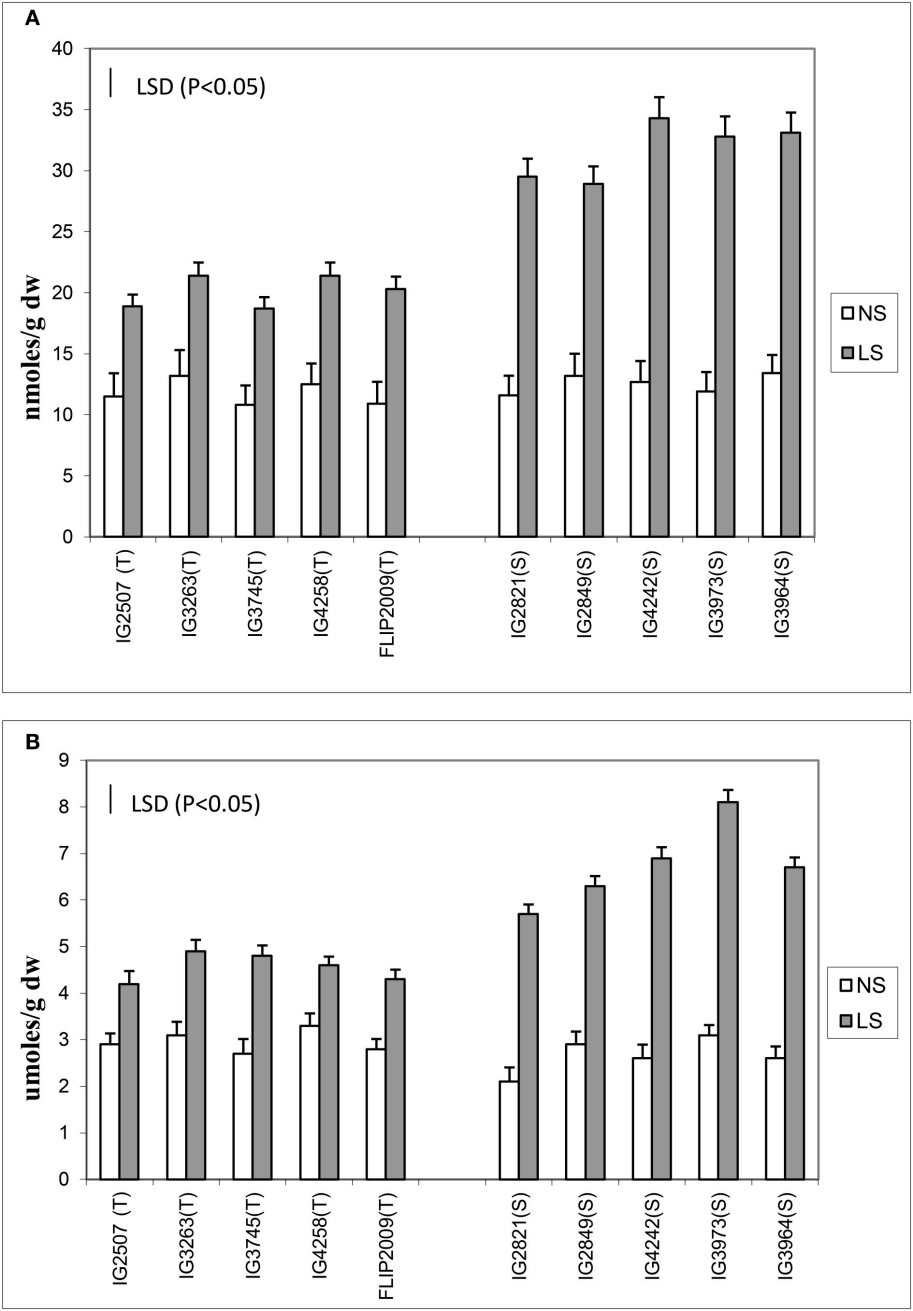
**(A)** Malondialdehyde (MDA) and **(B)** hydrogen peroxide (H_2_O_2_) concentration in normal-sown (NS) and late-sown (LS) lentil genotypes. Small vertical bars represent standard errors. LSD (*P* < 0.05; represented as small vertical line close to y-axis) for MDA (2.9) and H_2_O_2_ (0.83); T, tolerant; S, sensitive.

Oxidative damage was measured as malondialdehyde (MDA) and hydrogen peroxide concentration (Figure 3) in the leaves of NS and LS plants. MDA concentration is an indicator of oxidative damage to membranes, which was 1.6–2.7-fold higher in LS plants than in NS plants; HT genotypes showed significantly less damage (1.64–1.8-fold) than HS genotypes (2.1–2.7-fold). Hydrogen peroxide followed a similar trend in LS plants and showed 1.4–1.7-fold increase in HT genotypes and 2.1–2.8-fold increase in HS genotypes. Genotypes IG2507 and FLIP 2009 exhibited lowest damage as MDA and hydrogen peroxide concentrations, respectively.

**Figure 4:**
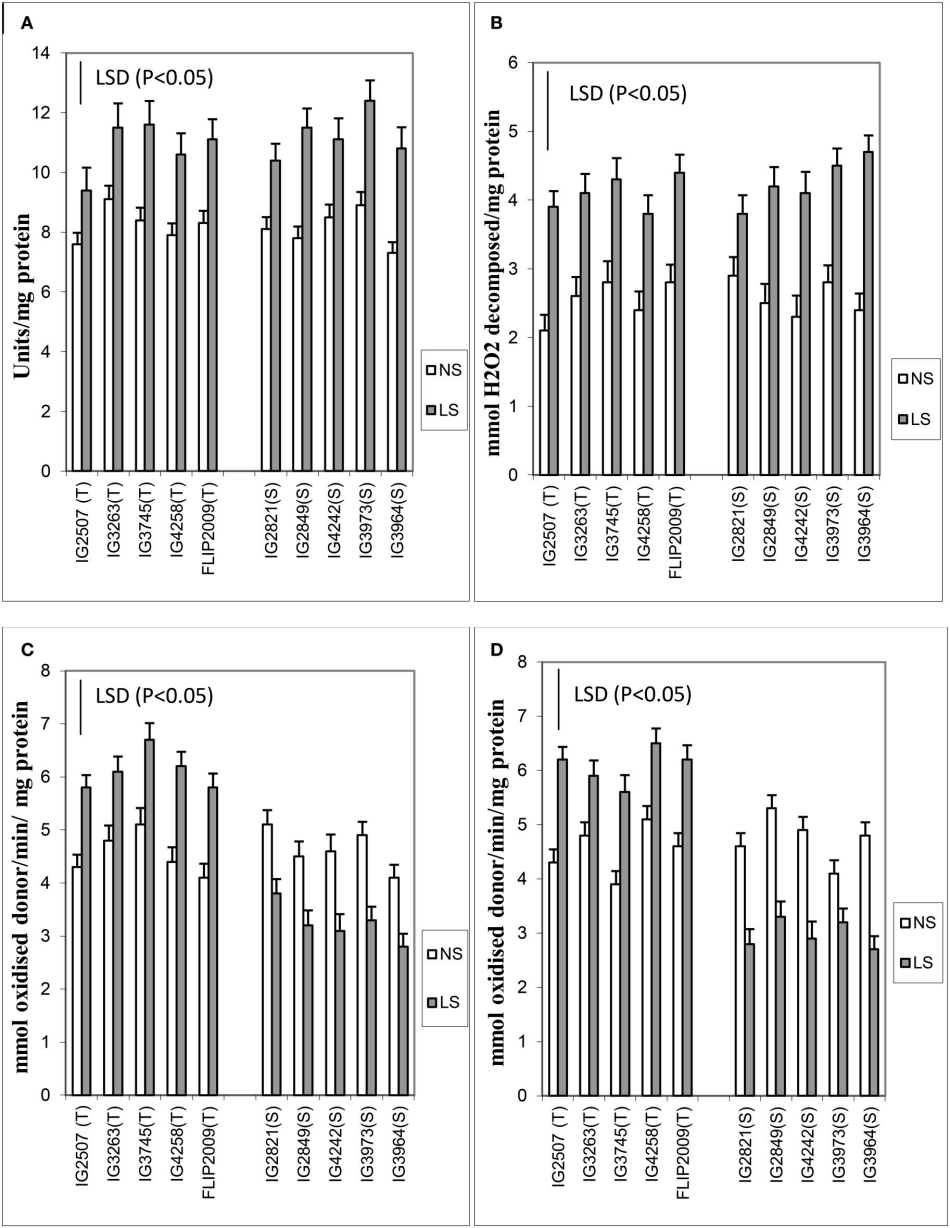
**(A)** Superoxide dismutase (SOD), **(B)** catalase (CAT), **(C)** ascorbate peroxidase (APX), and **(D)** glutathione reductase (GR) in normal-sown (NS) and late-sown (LS) genotypes. Small vertical bars on histograms represent standard errors. LSD (*P* < 0.05; represented as small vertical line close to y-axis) for SOD (1.2), CAT (1.6), APX (1.4), and GR (1.8); T, tolerant; S, sensitive.

Various antioxidants (enzymatic and non-enzymatic) were tested in leaves. Late-sown plants produced 25–47% more superoxide dismutase (SOD) than NS plants (Figure 4). Two HS genotypes (IG2849 and IG3964) produced significantly more SOD (47–48%) than the other genotypes. The differences between HT and HS genotypes for SOD activity were small.

Catalase increased more (31–95%) than SOD (25–47%) in LS plants (Figure 4). The genotypes IG2507 (HT) and IG3964 (HS) had the most enzyme activity while genotype IG2821 (HS) had the least.

Ascorbate peroxidase activity was 27–40% higher in LS plants of HT genotypes while it was 25–32% lesser in HS genotypes (Figure 4). Genotypes IG4258 (HT) and FLIP2009 (HT) produced the most APX under LS conditions.

Late-sown HT genotypes produced 23–44% more glutathione reductase than NS plants, while HS genotypes produced 22–43% less (Figure 4). GR activity showed highest increase in a HT genotype IG2507 under heat stress.

**Figure 5:**
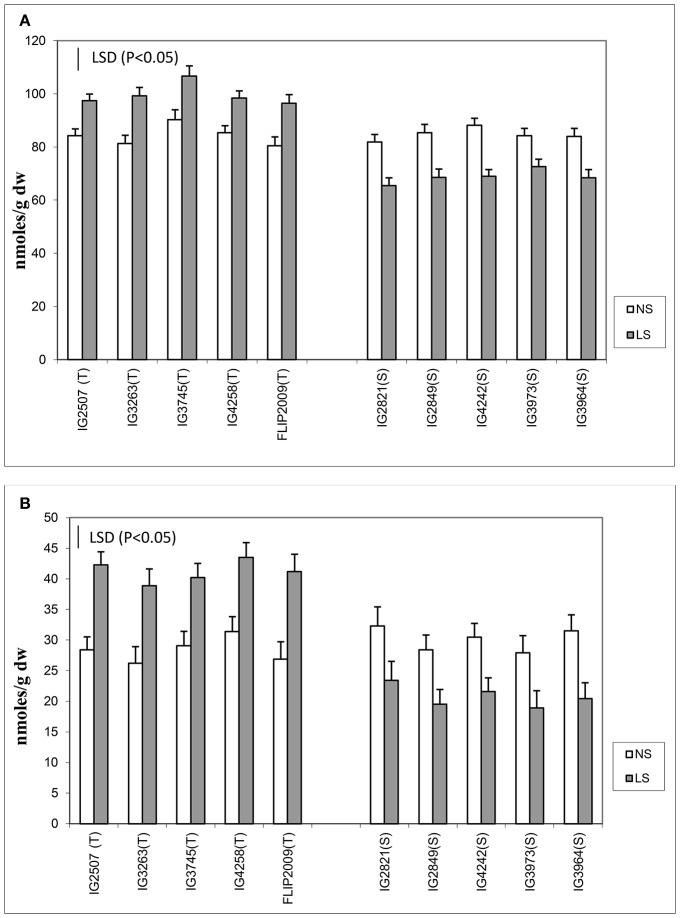
**(A)** Ascorbate (ASC) and **(B)** reduced glutathione (GSH) in normal-sown (NS) and late-sown (LS) genotypes. Small vertical bars on histograms represent standard errors. LSD (*P* < 0.05; represented as small vertical line close to y-axis) for ASC (4.7) and GSH (4.9); T, tolerant; S, sensitive.

Late-sown HT genotypes produced 15–22% more ascorbate (ASC) than NS plants, while it was found to be 13–21% less in HS genotypes (Figure 5). A HT genotype IG3745 had the highest increase in ASC concentration (22%) under LS conditions.

Reduced glutathione (GSH) concentration increased more than ASC concentration under heat stress. Late-sown HT genotypes produced 38–53% more GSH than NS plants, while HS genotypes produced 27–35% less (Figure 5). Highest GSH was found in Genotype IG3745 under heat stress environment.

### Sucrose and sucrose phosphate synthase activity in leaves and anthers

**Figure 6:**
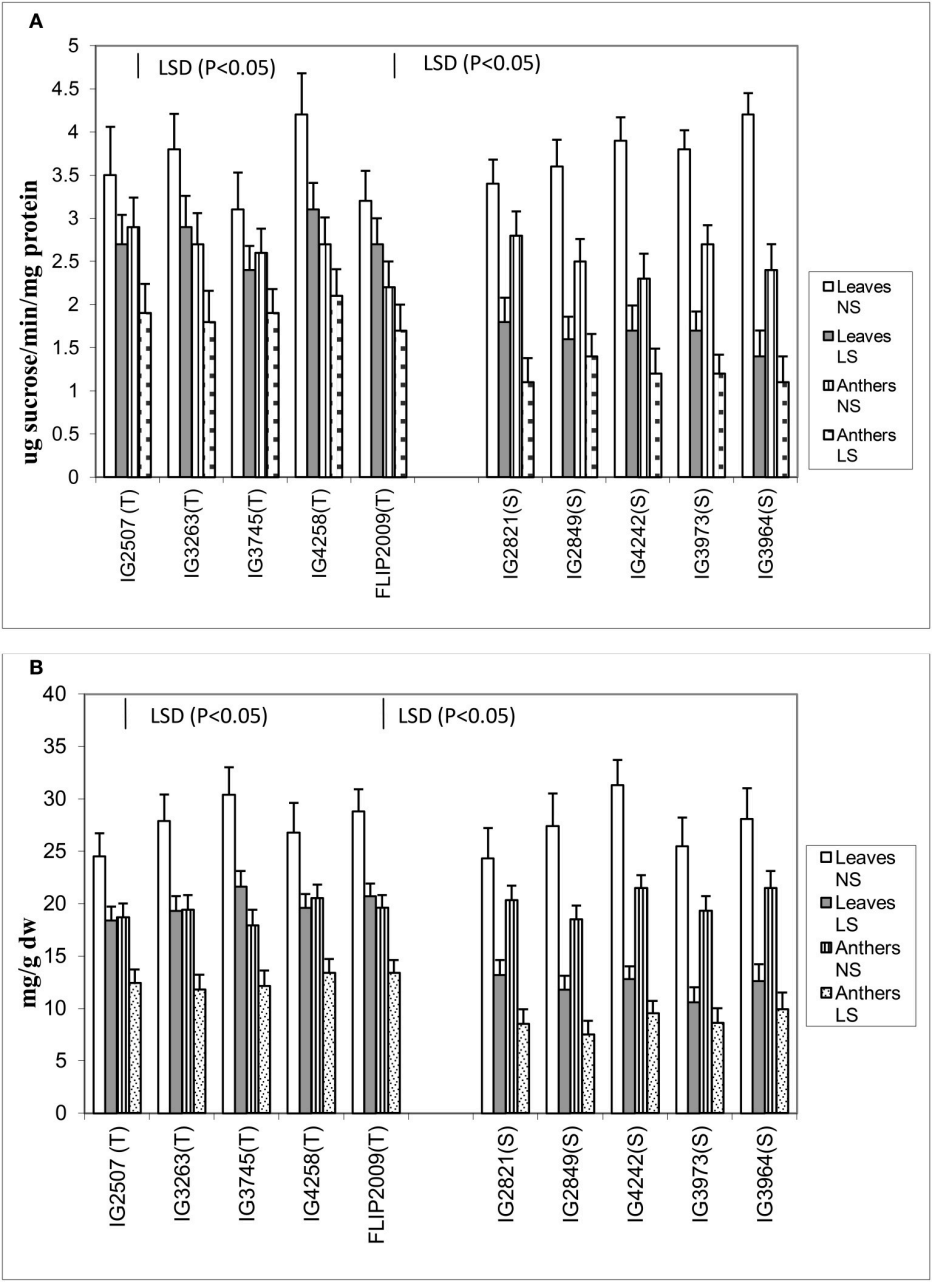
**(A)** Sucrose phosphate synthase (SPS) and **(B)** sucrose (SUC) in leaves and anthers of normal-sown (NS) and late-sown (LS) lentil genotypes. Small vertical bars represent standard errors. LSD (*P* < 0.05; represented as small vertical line close to y-axis) for SPS (leaves: 0.43; anthers: 0.39) and SUC (leaves:1.9, anthers: 1.7); T, tolerant; S, sensitive.

Late-sown plants significantly had less sucrose concentration in both the anthers and leaves than NS plants (Figure 6). Normal-sown plants had 24.3–31.3 mg g^−1^ DW sucrose in leaves and 17.9–21.5 mg g^−1^ DW in anthers compared with LS plants which had 10.6–21.6 mg g^−1^ DW in leaves and 7.5–13.4 mg g^−1^ DW in anthers. Late-sown HT genotypes had significantly higher sucrose concentrations in leaves (18.4–21.3 mg g^−1^ DW) and anthers (11.8–13.4 mg g^−1^ DW) than sensitive genotypes (leaves 10.6–13.2 mg g^−1^ DW; anthers 7.5–9.1 mg g^−1^ DW).

The leaves and anthers of NS plants had 3.1–4.2 units and 2.2–2.9 units of sucrose phosphate synthase (SPS) activity, respectively (Figure 6), but the activity was significantly less in LS plants (1.4–3.1 units in leaves; 1.1–2.2 units in anthers). SPS activity was significantly higher in HT genotypes, especially in the anthers, which had 50–75% more SPS activity than those of HS genotypes. No significant differences were noticed among the tolerant genotypes for sucrose concentration and SPS activity in leaves and anthers.

### Reproductive traits

**Figure 7:**
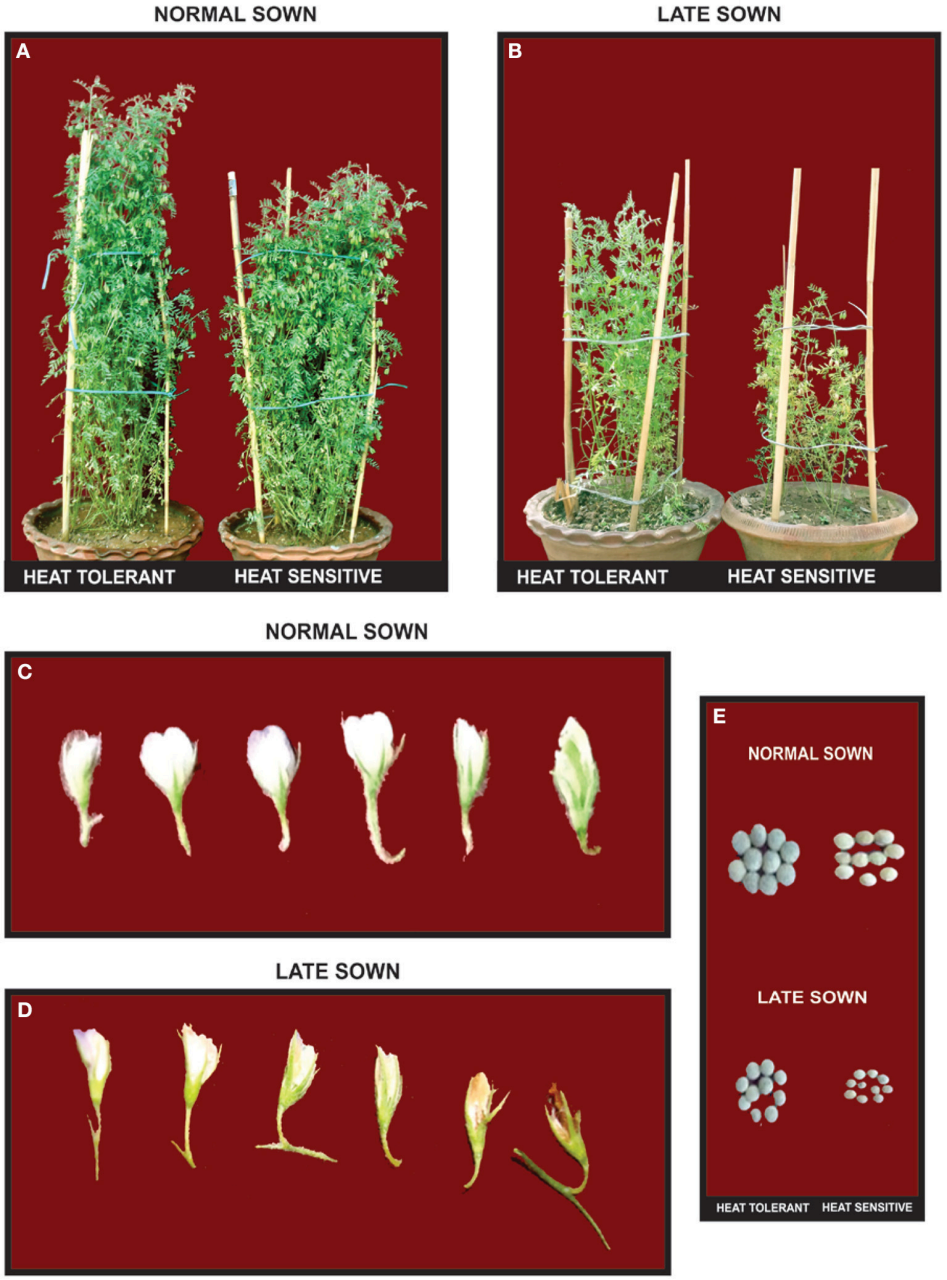
**Effects of normal sown (NS) and late sown (LS) environment in lentil genotypes grown in the field**. Biomass of **(A)** NS heat tolerant (HT) and heat sensitive (HS) genotypes, **(B)** LS (HT&HS genotypes); flowers of **(C)** NS and **(D)** LS plants; **(E)** seeds of NS and LS plants. Note the **(B)** reduced biomass in HS genotype, **(D)** damage to flowers, and **(E)** reduced seed size in LS plants.

**Figure 8:**
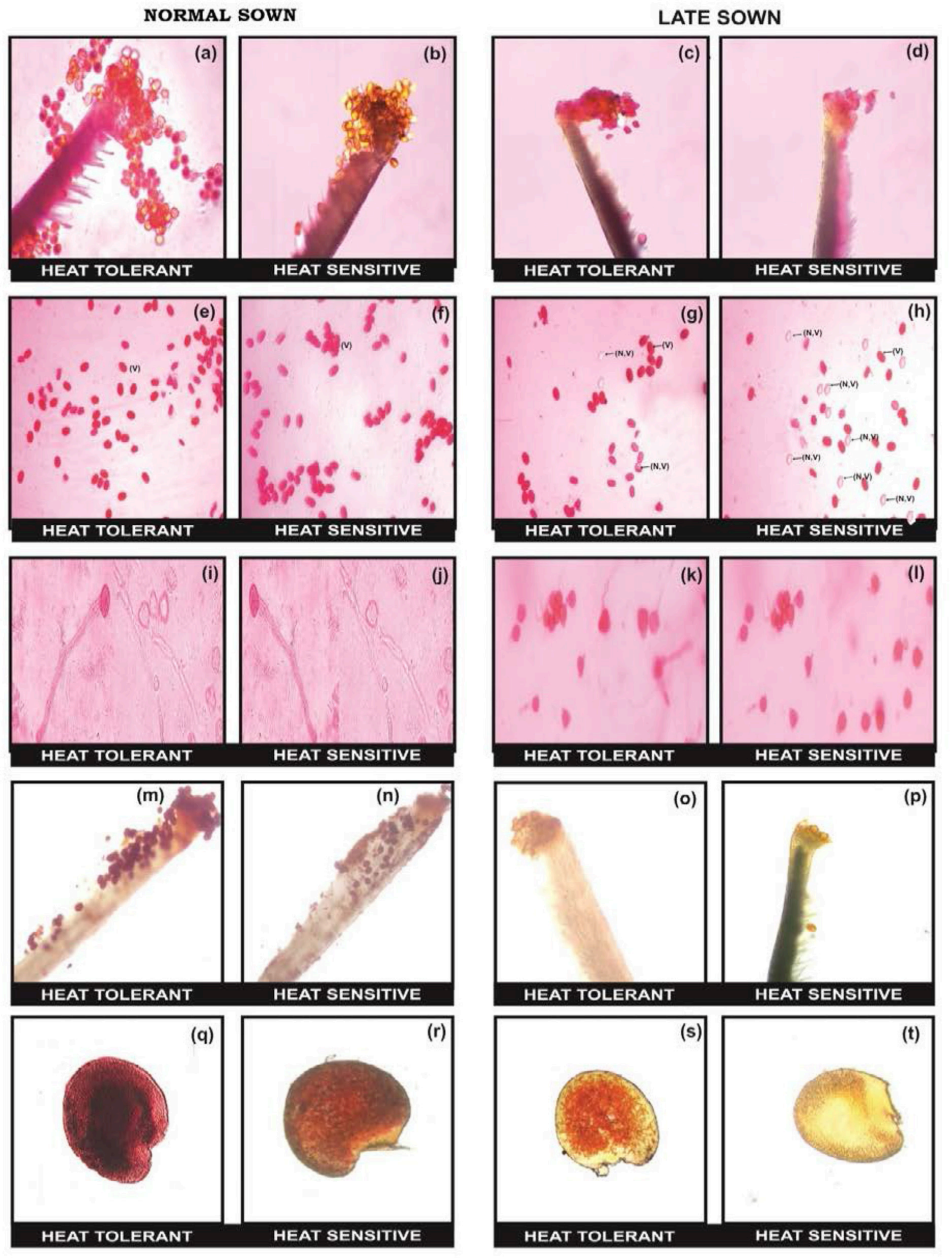
**Comparison of reproductive biology attributes in normal sown (NS) and late sown (LS) lentil plants**. Pollen load in **(a,b)** NS and **(c,d)** LS plants. Pollen viability [viable (V) and non-viable (NV) pollen grains] from **(e,f)** NS and **(g,h)** LS plants. *In vitro* pollen germination in **(i,j)** NS and **(k,l)** LS plants. Stigma receptivity in **(m,n)** NS and **(o,p)** LS plants. Ovule viability in **(q,r)** NS and **(s,t)** LS plants. Note marked reduction in **(d)** pollen load, **(h)** pollen viability, **(i)** pollen germination, **(p)** stigma receptivity, and **(t)** ovule viability in LS heat-sensitive genotypes compared with heat-tolerant genotypes.

**Figure 9:**
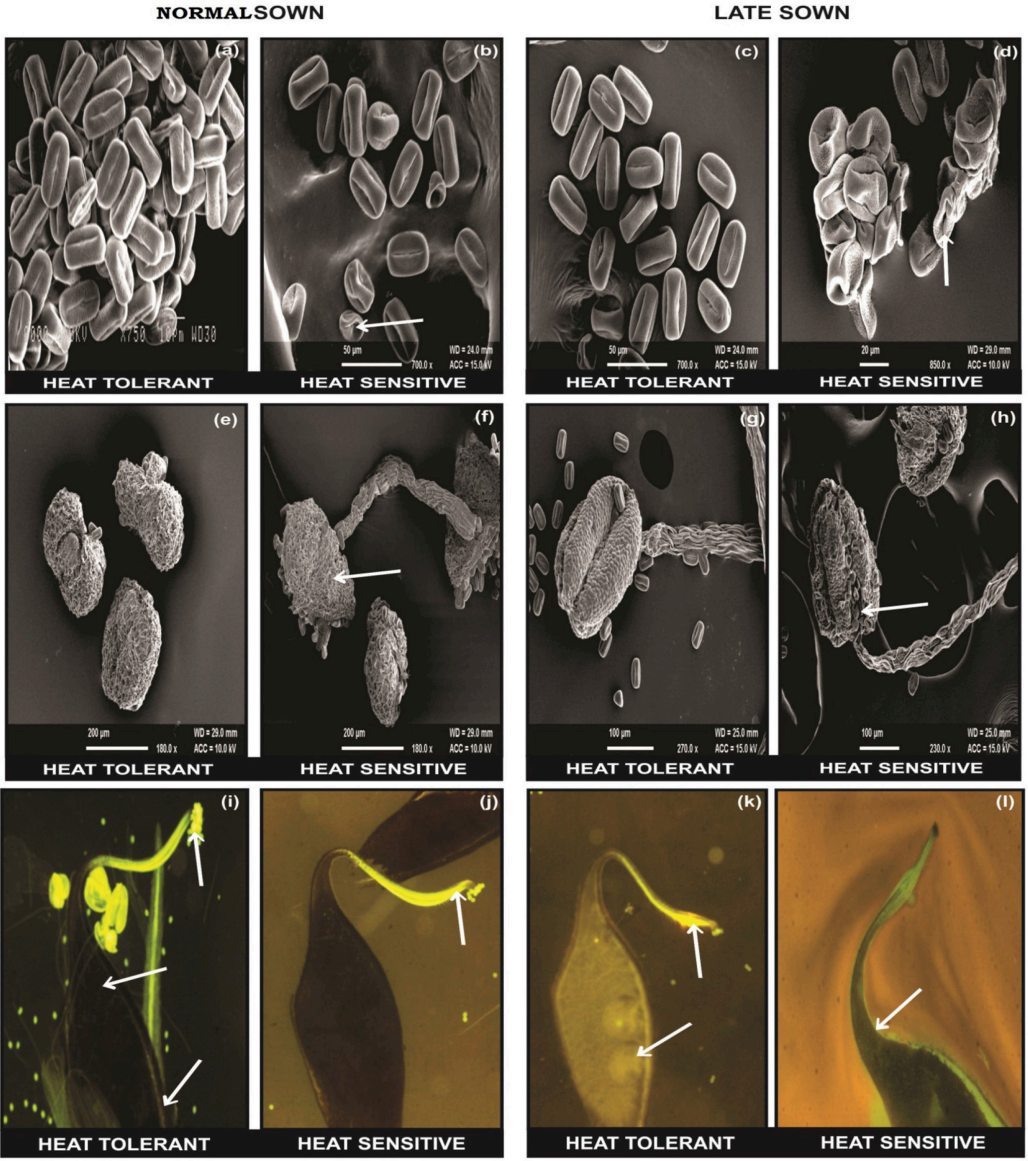
**SEM observations under normal (NS) and late-sown (LS) environment on reproductive components in lentil genotypes**. Pollen morphology in **(a,b)** NS and **(c,d)** LS plants. Anther morphology in **(e,f)** NS and **(g,h)** LS plants. Note damage to **(d)** pollen morphology and **(h)** anther morphology in LS heat-sensitive genotypes. Fluorescent microscopic studies showing the effects of heat stress on pollen load, pollen tube growth (marked as arrows) in the stylar region and ovular region in **(i,j)** NS and **(k,l)** LS plants. Note the **(l)** lack of pollen tube growth in the stylar region of LS heat sensitive genotypes compared with **(k)** heat-tolerant genotypes.

The flowers of LS plants showed significant damage to their morphology due to heat stress (Figure 7). The structure of anthers and pollen grains was adversely affected (Figures 8, 9). Late-sown plants had significantly less pollen viability (41.7–66.9%) than NS plants (78.4–83.4%) (Table 8). Under LS conditions, HT genotypes had more viable pollen (62.4–70.3%) than HS genotypes (40.9–45.3%) when late-sown. HT genotypes had 13.3–23.9% less pollen load compared with 36.6–42% less in HS genotypes (Table 8). *In vitro* pollen germination ranged from 70.3 to 78.2% in NS plants, but only 28.6–53.4% in LS plants (Table 8). Under heat stress, HT genotypes maintained 48–50% pollen germination compared to 28–33% in HS genotypes. In late-sown HS genotypes, pollen tube growth through the style was strongly inhibited (Figures 8, 9) unlike HT genotypes, which impaired fertilization and caused pod abortion. Genotype IG3263 was found to have highest pollen viability (70%) while pollen germination was maximum in IG3745 under heat stress environment.

**Table 8:** **Pollen function, stigma and ovular function in normal-sown (NS) and late-sown plants of heat-tolerant (HT) and heat-sensitive (HS) genotypes (Mean ± SE)**.

**Genotypes**	**Pollen viability (%)**		**Pollen load (1–5 scale)**		**Pollen germination (%)**		**Stigma receptivity (1–5 scale)**		**Ovule viability (1–5 scale)**	
	**NS**	**LS**	**NS**	**LS**	**NS**	**LS**	**NS**	**LS**	**NS**	**LS**
IG2507 (T)	83.4 ± 6.3a	64.7 ± 6.7a	4.6 ± 0.85a	3.5 ± 0.77a	73.4 ± 2.6a	47.9 ± 4.1b	4.6 ± 0.81a	3.5 ± 0.67a	4.3 ± 0.67a	3.1 ± 0.71a
IG3263(T)	81.4 ± 6.1a	70.3 ± 6.3a	4.8 ± 0.78a	3.8 ± 0.71a	77.9 ± 2.8a	45.3 ± 3.8b	4.6 ± 0.76a	3.4 ± 0.71a	4.2 ± 0.71a	3.3 ± 0.70a
IG3745(T)	82.5 ± 6.8a	66.1 ± 5.9a	4.9 ± 0.67a	3.8 ± 0.63a	78.2 ± 3.1a	53.4 ± 3.3a	4.8 ± 0.72a	3.6 ± 0.72a	4.5 ± 0.68a	3.7 ± 0.78a
IG4258(T)	80.3 ± 6.3a	62.4 ± 6.1a	4.6 ± 0.71a	3.8 ± 0.61a	75.3 ± 2.9a	43.4 ± 2.8b	4.6 ± 0.79a	3.6 ± 0.73	4.7 ± 0.69a	3.3 ± 0.73a
FLIP2009(T)	81.6 ± 5.8a	63.9 ± 7.3a	4.5 ± 0.82a	3.9 ± 0.58a	71.3 ± 3.3a	50.3 ± 3.2a	4.8 ± 0.80a	3.8 ± 0.70a	4.2 ± 0.71a	3.4 ± 0.72a
IG2821(S)	81.8 ± 7.3a	44.8 ± 5.9b	4.7 ± 0.69a	2.6 ± 0.58b	72.4 ± 3.7a	31.4 ± 2.8c	4.2 ± 0.75a	2.1 ± 0.70b	4.6 ± 0.72a	2.3 ± 0.71b
IG2849(S)	83.2 ± 6.4a	40.9 ± 6.1b	4.1 ± 0.72a	2.4 ± 0.6b	70.3 ± 3.8a	28.6c ± 3.1	4.4 ± 0.71a	2.3 ± 0.73b	4.8 ± 0.71a	2.1 ± 0.73b
IG4242(S)	79.5 ± 5.9a	41.7 ± 4.6b	4.3 ± 0.74a	2.5 ± 0.71b	73.4 ± 4.1a	30.8 ± 2.8c	4.1 ± 0.72a	2.1 ± 0.76b	4.4 ± 0.73a	2.3 ± 0.74b
IG3973 (S)	80.1 ± 6.1a	45.3 ± 3.6b	4.5 ± 0.81a	2.3 ± 0.70b	78.1 ± 3.8a	36.4 ± 2.6c	4.3 ± 0.79a	2.5 ± 0.72b	4.8 ± 0.74a	2.3 ± 0.67b
IG3964 (S)	78.4 ± 5.9a	42.9 ± 3.8b	4.7 ± 0.83a	2.4 ± 0.58b	73.4 ± 3.9a	33.2 ± 2.4c	4.2 ± 0.71a	2.3 ± 0.71b	4.4 ± 0.75a	2.1 ± 0.61b
LSD (*P* < 0.05) (genotypes × sowing interaction)	8.1		0.85		4.1		0.83		0.81	

*Similar letters in a vertical column indicate no significant difference from each other. LSD, least significant difference*.

Late-sown plants had 20–51% less stigma receptivity than NS plants (Table 8) but was less inhibited in HT genotypes (20–26%) than in HS genotypes (41–51%). Similarly, LS plants had less ovule viability (19–56%) than NS plants, with less damage to HT genotypes (19–27%) than to HS genotypes (47–56%) (Table 8). No significant differences were observed for stigma receptivity and ovule viability among different tolerant genotypes under heat stress environment.

Late-sown HS genotypes had impaired pollen tube growth through the style leading to failure of fertilization (Figures 8, 9) due to heat stress, while the pollen tubes in HT genotypes were clearly visible suggesting a normal fertilization process.

Pollen grains harvested from flowers of NS and LS plants were grown in a sucrose-supplemented medium (1.0 and 2.5 μM). Germination of pollen grains collected from flowers of LS plants, especially those from sensitive genotypes, improved significantly with sucrose supplementation compared to untreated pollen grains (Table 9).

**Table 9:** **Effect of sucrose supplementation in growth medium on pollen germination (%) in tolerant (T) and sensitive (S) germination (Mean ± SE)**.

**Genotypes**	**Control**	**1 uM**	**2.5 UM**
IG3745 (T)	58.9 ± 5.9a	65.8 ± 4.9a	68.4 ± 5.3a
FLIP2009 (T)	61.3 ± 5.8a	78.4 ± 5.1a	76.9 ± 6.1a
IG2849 (S)	35.7 ± 4.7b	60.5 ± 6.0ab	62.4 ± 5.4ab
IG4242(S)	40.3 ± 5.3b	63.2 ± 5.4ab	64.3 ± 5.2ab
LSD (*P* < 0.05) (genotypes × treatment Interaction)	7.3		

*Similar letters in a vertical column indicate no significant difference from each other. LSD, least significant difference*.

### Controlled environment studies

Two HT genotypes (IG3745, FLIP2009) and two HS genotypes (IG2849, IG4242) were subjected to heat stress (35/25°C, 38/28°C, 40/30°C) at flowering stage in a controlled-environment growth chamber and data for various traits recorded to validate observations from the outdoor experiment.

Compared to the plants grown at 25/15°C (control), the number of pods/plant in HT genotypes exposed to 35/25°C decreased by 29–35% and 48–52% in HS genotypes (Table 10). The pods decreased further with increasing temperature; by 38–39% and 76–81% at 38/28°C and 40/30°C, respectively, in HT genotypes while no pods were formed in HS genotypes at these temperatures.

**Table 10:** **Effect of varying heat stress on pods and seed weight/plant in heat-tolerant (T) and heat-sensitive (S) genotypes grown under controlled environment**.

**Temperature**	**Pods/plant**	**Seed weight/plant**
**25/15**°**C**		
IG3745 (T)	117.4 ± 6.9a	3.98 ± 0.67a
FLIP2009 (T)	123.4 ± 7.1a	4.11 ± 0.73a
IG2849(S)	80.5 ± 7.7c	2.84 ± 0.76b
IG4242(S)	78.4 ± 6.0d	2.76 ± 0.72b
**35/25**°**C**		
IG3745(T)	89.4 ± 7.3b	2.56 ± 0.58b
FLIP2009(T)	93.2 ± 6.8b	2.88 ± 0.61b
IG2849(S)	38.5 ± 5.8e	1.13 ± 0.56c
IG4242(S)	40.4 ± 4.7e	1.02 ± 0.34c
**38/28**°**C**		
IG3745 (T)	72.4 ± 6.7c	1.56 ± 0.34c
FLIP2009 (T)	75.2 ± 7.2c	1.43 ± 0.42c
IG2849(S)	0	0
IG4242(S)	0	0
**40/30**°**C**		
IG3745(T)	27.5 ± 6.9f	0.78 ± 0.21d
FLIP2009(T)	22.6 ± 6.1f	0.64 ± 0.25d
IG2849(S)	0	0
IG4242(S)	0	0
LSD (*P* < 0.05) (genotypes × treatment Interaction)	8.2	0.79

*The plants were initially grown under outdoor conditions and moved to controlled environment at the time of flowering initiation (Mean ± SE). Similar letters in a vertical column indicate no significant difference from each other. LSD, least significant difference*.

Seed weight per plant in HT and HS genotypes grown at 35/25°C was 29–35% and 61–63%, respectively, less than the control plants (Table 10). At 38/28°C, HT genotypes had 62–65% less seed weight, while HS genotypes did not produce any seeds.

Pollen viability and germination were inhibited strongly in sensitive genotypes at 35/25°C (Table 11). At higher temperatures, HS genotypes had few viable pollen grains and no germination, while pollen grains of HT genotypes maintained some viability and germination. Similarly, stigma receptivity and ovule viability were severely affected in sensitive genotypes even at 35/25°C. At 38/28°C and 40/30°C, these traits were severely affected in sensitive genotypes due to flower damage, while tolerant genotypes retained stigma and ovular function, though to a lesser extent.

**Table 11:** **Effect of varying heat stress on pollen function, stigma and ovular viability in heat-tolerant (T) and heat-sensitive (S) genotypes grown under controlled environment (Mean ± SE)**.

**Temperature**	**Pollen viability (%)**	**Pollen germination (%)**	**Stigma receptivity (1–5 scale)**	**Ovule viability (1–5 scale)**
**25/15**°**C**				
IG3745 (T)	78.3 ± 7.8a	81.2 ± 7.8a	4.3 ± 0.56a	4.6 ± 0.37a
FLIP2009 (T)	81.3 ± 8.2a	76.8 ± 8.2a	4.6 ± 0.63a	4.4 ± 0.49a
IG2849(S)	76.9 ± 8.8a	74.9 ± 8.9a	4.2 ± 0.67a	4.4 ± 0.31a
IG4242(S)	82.4 ± 7.5a	83.5 ± 7.1a	4.6 ± 0.61a	4.2 ± 0.49a
**35/25**°**C**				
IG3745(T)	67.4 ± 8.2b	60.6 ± 6.8b	3.9 ± 0.51a	3.8 ± 0.31b
FLIP2009(T)	72.4 ± 7.6b	68.6 ± 7.2b	3.6 ± 0.58a	3.2 ± 0.43b
IG2849(S)	56.3 ± 5.8bc	43.4 ± 4.7c	1.8 ± 0.35c	1.5 ± 0.38d
IG4242(S)	43.2 ± 4.8c	39.6 ± 3.8c	1.4 ± 0.31c	1.9 ± 0.48d
**38/28**°**C**				
IG3745(T)	61.5 ± 6.8b	53.5 ± 5.7b	2.7 ± 0.16b	2.8 ± 0.33c
FLIP2009(T)	68.3 ± 7.3b	58.9 ± 6.4b	2.9 ± 0.18b	2.6 ± 0.48c
IG2849(S)	26.6 ± 3.3e	0	0	0
IG4242(S)	18.9 ± 3.1e	0	0	0
**40/30**°**C**				
IG3745(T)	35.6 ± 2.5cd	32.4 ± 3.7cd	1.3 ± 0.15c	1.4 ± 0.32d
FLIP2009(T)	38.2 ± 2.7cd	35.6 ± 4.3cd	1.5 ± 0.17c	1.5 ± 0.45d
IG2849(S)	0	0	0	0
IG4242(S)	0	0	0	0
LSD (*P* < 0.05) (genotypes × treatment Interaction)	8.2	8.9	0.67	0.57

*Similar letters in a vertical column indicate no significant difference from each other. LSD, least significant difference*.

Membrane damage (as electrolyte leakage) increased markedly with increasing temperatures (Table 12). HS genotypes showed more injury (>30%) at 38/28 and 40/30°C than HT genotypes (<20% injury).

**Table 12:** **Electrolyte leakage (EL), relative leaf water content (RLWC), chlorophyll (Chl), Photosystem (PS) II function and stomatal conductance (gS) in heat-tolerant (T) and heat-sensitive (S) genotypes grown under controlled environment (Mean ± SE)**.

**Temperature**	**EL (%)**	**RLWC (%)**	**Chl (mg/g dw)**	**PS II (Fv/Fm ratio)**	**gS (m mol/m^−2^/s^−1^)**
**25/15**°**C**					
IG3745 (T)	8.9 ± 1.4a	83.4 ± 5.7a	14.9 ± 1.4a	0.8 ± 0.056a	324.5 ± 12.5b
FLIP2009 (T)	7.6 ± 1.8a	84.6 ± 5.9a	16.7 ± 1.6a	0.8 ± 0.061a	318.9 ± 14.8b
IG2849(S)	6.9 ± 1.7e	82.6 ± 6.1a	14.6 ± 1.3a	0.8 ± 0.065a	345.6 ± 15.8b
IG4242(S)	9.9 ± 1.6e	84.1 ± 5.8a	16.1 ± 1.3a	0.8 ± 0.064a	318.5 ± 16.8b
**35/25**°**C**					
IG3745(T)	14.5 ± 1.8cd	76.4 ± 6.3a	13.2 ± 1.8a	0.76 ± 0.061a	398.5 ± 18.4b
FLIP2009(T)	12.8 ± 1.9cd	78.9 ± 6.9a	15.3 ± 1.7a	0.72 ± 0.068ab	410.7 ± 0.19.1a
IG2849(S)	23.5 ± 1.8c	63.2 ± 5.8b	9.8 ± 1.3cd	0.61 ± 0.063b	267.9 ± 17.9d
IG4242(S)	21.8 ± 1.8c	60.5 ± 6.2b	8.3 ± 1.4d	0.56 ± 0.061bc	275.4 ± 18.1d
**38/28**°**C**					
IG3745(T)	17.3 ± 1.6cd	71.3 ± 6.6a	11.2 ± 1.2bc	0.7 ± 0.061b	401.3 ± 15.9a
FLIP2009(T)	19.5 ± 2.2c	68.4 ± 5.9b	12.5 ± 1.4b	0.64 ± 0.068b	413.4 ± 17.3a
IG2849(S)	30.2 ± 2.7b	53.2 ± 6.3d	8.2 ± 1.1d	0.43 ± 0.069d	220.4 ± 18.1e
IG4242(S)	30.8 ± 2.9b	56.2 ± 5.8d	7.3 ± 1.1d	0.4 ± 0.064d	224.6 ± 19.3e
**40/30**°**C**					
IG3745(T)	20.5 ± 2.2c	60.4 ± 5.4c	8.9 ± 1.3d	0.58 ± 0.066bc	298.5 ± 20.5c
FLIP2009(T)	22.5 ± 2.5c	60.8 ± 4.9c	9.3 ± 1.5cd	0.61 ± 0.069b	280.6 ± 22.4c
IG2849(S)	34.5 ± 3.1a	40.3 ± 4.1e	5.6 ± 1.4e	0.32 ± 0.060e	71.9 ± 24.5f
IG4242(S)	35.8 ± 3.4a	42.2 ± 4.5e	4.8 ± 1.1e	0.28 ± 0.063e	76.3 ± 27.8f
LSD (*P* < 0.05) (genotypes × treatment Interaction)	3.7	7.4	1.8	0.070	26.6

*Similar letters in a vertical column indicate no significant difference from each other. LSD, least significant difference*.

Water status, measured as RLWC, decreased with increasing temperature more so in HS genotypes than HT genotypes (Table 12). For example, compared to control (25/15°C), RLWC decreased to 53–56% in HS genotypes at 38/28°C while HT genotypes retained higher values (68–71%) at this temperature.

Stomatal conductance (*gS*) increased in HT genotypes with increasing temperature up to 38/28°C but decreased, remarkably in HS genotypes at all the heat stress (Table 12).

The leaves in HS genotypes had more chlorosis than HT genotypes at all the higher temperatures due to a severe reduction in total chlorophyll (Table 12). At higher temperatures (38/28 and 40/30°C), HS genotypes exhibited 40–50% more chlorophyll loss than HT genotypes.

Photosynthetic efficiency (as PSII function) declined more in HS genotypes at all higher temperatures. For example, at 38/28°C, in HT genotypes, it decreased by 12–13% while in HS genotypes, it declined by 46–50%, compared with the control (25/15°C).

## Discussion

We exposed 38 lentil genotypes to heat stress during the reproductive stage by sowing them outdoors 2 months later than recommended. As a result, LS plants experienced the impact of higher temperatures [>35/20°C; RH: 93–42% (max; day time)/59–12% (min; night time)] during the reproductive stage compared with NS plants [>35/20°C; RH: 95–77% (max; day time)/61–14% (min; night time)]. Typically, sowing crops later in the growing season is a technique used for screening genotypes for high-temperature tolerance, e.g., chickpea (Krishnamurthy et al., 2011; Upadhyaya et al., 2011; Awasthi et al., 2014) and mungbean (Sharma et al., 2016). Since high temperature stress combined with low RH increase the rate of transpiration and may generate water stress in a late-sown environment, the plants were frequently watered to avoid any confounding effects of dehydration. Nevertheless, the relative leaf water content in LS plants declined significantly, especially in HS genotypes, an indication of water deficit stress which added to the effects of the high-temperature stress.

There was a marked reduction in biomass, pods, seed yield and 100-seed weight in LS plants due to heat stress. Observations on these traits revealed that genotypes which maintained relatively higher biomass, seed yield and 100-seed weight under heat stress could be termed heat-tolerant (IG2507, IG3263, IG3297, IG3312, IG3327, IG3546, IG3330, IG3745, IG4258, and FLIP2009) while those with low values of these traits were termed heat-sensitive (IG2506, IG2519, IG2802, IG2821, IG2849, IG2878, IG3290, IG3326, IG3568, IG3973, IG3964, IG4221, IG4242, DP315, and DPL15).

Based on this screening, we selected five HT genotypes (IG2507, IG3263, IG3745, IG4258, and FLIP2009) and five HS genotypes (IG2821, IG2849, IG3973, IG3964, and IG4242) for subsequent studies to determine the mechanisms related to heat tolerance. Biomass decreased markedly in LS plants due to heat stress which inhibited vegetative growth, hastened reproductive growth, and reduced flower and pod numbers, which reduced seed yield markedly, more so in HS genotypes. The production of fewer flowers appeared to be more detrimental in influencing pod number than pod set, though the latter also contributed markedly toward decrease in yield. Similar effects of high temperature stress have been found in some other legume crops too, for example, chickpea (Kaushal et al., 2013) and mungbean (Kaur et al., 2015). A drastic reduction in the duration of flowering and podding appeared to reduce pod numbers and seed yield in the present study, which agrees with previous findings on other crops (Kaushal et al., 2013; Prasad et al., 2017).

Nodule numbers decreased under heat stress in all genotypes, more so in HS genotypes than HT genotypes. The reduction in nodule number agrees with a previous study on soybean (Keerio et al., 2001), where nitrogenase activity also decreased. In our study, the inhibition of sucrose production in leaves due to heat stress might have affected its supply for nodule formation, which needs to be examined further in nodules. HT genotypes produced more nodules, which is in accordance with earlier findings (Keerio, 2001), and may be attributed to their ability to produce more sucrose in leaves under heat stress, as we found in the present study.

Heat stress damaged the leaves causing chlorosis, scorching, and mild to severe burning of leaves, which affected the overall performance of plants by inhibiting flower production and pod set which substantially reduced seed yield. These reports are matching some previous findings on chickpea (Kumar et al., 2013) and mungbean (Kaur et al., 2015). Our results indicated that the leaves of LS plants under heat stress were adversely affected and responded by increasing their temperature, reducing leaf water status and inhibiting stomatal conductance, which agrees with previous reports on other crops (Prasad et al., 2017). Heat stress results in decrease in water content of the cells, reducing cell size and, in due course, restricting plant growth (Rodriguez et al., 2005; Sharma et al., 2016). Heat negatively impacts leaves by reducing leaf water potential, reducing leaf area and causing premature leaf senescence, all of which affect a plant's total photosynthesis performance (Greer and Weedon, 2012; Sharma et al., 2016). Consequently, heat stress increased membrane damage, which inhibited photosynthetic function and chlorophyll concentration, a finding similar to heat-stressed plants in chickpea (Kaushal et al., 2013). Leaf damage under heat stress correlated with increased oxidative stress indicators (malondialdehyde and hydrogen peroxide) and reduced antioxidative capacity, particularly in HS genotypes, which correlates with earlier findings in mungbean (Kumar et al., 2011).

Sucrose production in the leaves and anthers is vital for their function. In LS plants, sucrose concentration declined markedly in both organs due to heat stress, which correlated with an inhibition of the activity of the sucrose-synthesizing enzyme sucrose phosphate synthase. Heat stress negatively affects sucrose metabolism due to the inhibition of carbon fixation and assimilation (Awasthi et al., 2015) to influence seed yield. Reductions in sucrose concentrations in the leaves and anthers exposed to heat stress may be linked to decrease in RuBisCo activity (or increased photorespiration) and sucrose-synthesizing enzymes (Ray et al., 2003; Awasthi et al., 2014). “Shortened growth (biomass) may be due to reduction in leaf expansion and elongation and, consequently, the yield-contributing components (flowers, pods and seeds), which require leaves to obtain sucrose as well as other macromolecules” (Hanumantha Rao et al., 2016). Therefore, protection of the photosynthetic mechanism of leaves is imperative at the time of exposure to supra-optimal temperature to continue the synthesis and transport of sucrose to pods and seeds (Awasthi et al., 2014; Hanumantha Rao et al., 2016).

Our study highlights a strong negative impact of high temperature on reproductive function in genotypes of lentil, which could be due to the direct effects of heat stress on floral organs or to a marked reduction in sucrose synthesis and/or transport to flowers (Kaushal et al., 2013), aggravated by chlorotic leaf tissue damage. These impairments are likely to hamper the function of floral components and the development of new flowers; causing less pods and seeds, as well as small-sized seeds. In the present study, heat stress drastically decreased pollen viability, pollen germination and pollen tube growth in the style, which may have impaired fertilization leading to flower abortion (Kaushal et al., 2013; Prasad et al., 2017). This was associated with loss of stigma receptivity and ovule viability, which contributed toward loss of flower fertility, and the number of filled pods. Flower function requires the transport or synthesis of optimum sucrose concentrations, which were inhibited in leaves and anthers of lentil in our study due to its reduced synthesis resulting in floral abortion.

Pod set relies on the performance of the male and female components of flowers that are susceptible to high temperature stress in crops like cotton (Snider et al., 2009), chickpea (Kumar et al., 2013), tomato (Li et al., 2012), and mungbean (Kaur et al., 2015). The reduction in pod set as a result of heat stress in LS plants in our study is similar to observations in soybean (Djanaguiraman et al., 2013), chickpea (Kaushal et al., 2013), and mungbean (Kaur et al., 2015). The restricted availability and transport of sucrose have been implicated in reduced pod numbers in heat-stressed plants (Kaur et al., 2015). Moreover, pods and seeds were smaller in heat-stressed lentil plants than the controls, which indicated a reduction in sucrose translocation to developing seeds (Li et al., 2012; Awasthi et al., 2014). Measuring sucrose concentration in pods and seeds may provide better insight and shall be part of our next study on this aspect.

### Putative mechanisms of heat tolerance

Observations on contrasting genotypes revealed that leaf temperature did not rise appreciably in tolerant genotypes under heat stress, compared with sensitive genotypes, which protected the plants from the adverse effects of heat stress. This was attributed to higher stomatal conductance and relative leaf water content resulting in transpirational cooling and matches observations in tolerant genotypes of other crop species (Annisa et al., 2013; Awasthi et al., 2014). Consequently, tolerant genotypes had much less leaf damage, in terms of tissue electrolyte leakage, PSII function, chlorophyll concentration and cellular oxidizing ability. Moreover, the anthers and leaves of tolerant genotypes had higher sucrose concentrations, possibly due to less damage, which contributed toward the production of more biomass, flowers, pods and seeds under heat stress, compared with sensitive genotypes. In some previous studies, sucrose production and utilization was associated with the ability to yield better under heat stress (Awasthi et al., 2014). The reproductive function was also superior in HT genotypes, which correlated with higher sucrose concentrations and SPS activity in leaves and anthers. Moreover, improved germination of pollen grains from heat-stressed plants, by the exogenous application of sucrose, further linked the possibility of sucrose depletion from anthers as a reason for the loss of pollen function (Bita and Gerats, 2013).

One adverse effect of heat stress is increased production of ROS and hydrogen peroxide, which may damage membranes and macromolecules to severely inhibit growth (Awasthi et al., 2015). The leaves of HT genotypes suffered less oxidative damage than HS genotypes, which was associated with higher expression of antioxidants, especially the components of the ascorbate–glutathione cycle (ascrobate peroxidase, glutathione reductase, ascrobate and reduced glutathione), to scavenge ROS and hydrogen peroxide (Hasanuzzaman et al., 2013). Thus, our findings in this regard in contrasting lentil genotypes are in agreement with observations in some other crop species, including brassica (Wilson et al., 2014), Kentucky bluegrass (*Poa pratensis L*.) (Li et al., 2014), and chickpea (Kaushal et al., 2013). Though, there were variations in oxidative defense in neutralizing hydrogen peroxide in some genotypes, considering the higher expression of components of ascorbate/glutathione pathway in HT genotypes, the heat tolerance in the present study might be partly related to this pathway. This concurs with previous studies on cereals (Kumar et al., 2012) and chickpea (Kumar et al., 2013) subjected to heat stress.

### Controlled environment studies

Controlled environment (CE) studies were performed to confirm the findings from the outdoor studies and to quantify the stressful temperatures. These studies validated the adverse effect of high temperature at the time of flowering on various components of yield, which was associated with leaf damage in terms of reduced membrane integrity, loss of chlorophyll and PSII function. While HT genotypes selected from our screening studies were partly able to bear temperatures up to 40/30°C, HS genotypes were severely affected even at 35/25°C and failed to produce any pods at further temperatures. This was associated with the superior reproductive function of tolerant genotypes at all the tested temperatures, compared with the sensitive genotypes. The leaves of HT genotypes also suffered less damage in terms of membrane injury, chlorosis and photosynthetic function, which was ascribed to the maintenance of higher stomatal conductance and hence leaf water content, relative to HS genotypes. Our observations are supported by previous similar studies on contrasting genotypes of chickpea (Kumar et al., 2013), brassica (Wilson et al., 2014), and wheat (Sairam et al., 2000; Dhyani et al., 2013) where heat-tolerant lines suffered less damage to their leaves and reproductive function (Devasirvatham et al., 2012). CE studies clearly demonstrated that temperatures >35/25°C would be highly detrimental for lentil genotypes

## Conclusions

Our study identified some heat-tolerant lentil genotypes (IG2507, IG3263, IG3297, IG3312, IG3327, IG3546, IG3330, IG3745, IG4258, and FLIP2009), which could be used as potential parents in a breeding program. In contrast, genotypes IG2821, IG2849, IG4242, IG3973, IG3964 were found to be most heat-sensitive. Studies in outdoor and controlled environments on HT and HS lentil genotypes indicated that heat tolerance in lentil was mainly determined by pollen function, which depended upon the availability of sucrose to anthers and pollen grains. Moreover, tolerant genotypes exhibited multiple tolerance mechanisms such as the protection of leaf tissues by limiting oxidative damage, which consequently maintained optimum photosynthetic and respiratory function, and produced more nodules.

## Author contributions

The experimental work was done by KS and AS. The germplasm was provided by JK, SK, and SS. The data analysis and Ms. was written by HN. The Ms. was edited by KHMS.

## Funding

The authors are thankful to UGC, DST New Delhi, ICARDA, Morocco for financial assistance to carry out this work.

### Conflict of interest statement

The authors declare that the research was conducted in the absence of any commercial or financial relationships that could be construed as a potential conflict of interest.
